# Supplementary material for: Paclitaxel binds and activates C5aR1: A new potential therapeutic target for the prevention of chemotherapy-induced peripheral neuropathy and hypersensitivity reactions
Source: Cell Death Dis. 2022 May 25;13(5):500. doi: 10.1038/s41419-022-04964-w (PMC9130998; doi:10.1038/s41419-022-04964-w)

Full and uncropped western blot for FIGURE 3

A

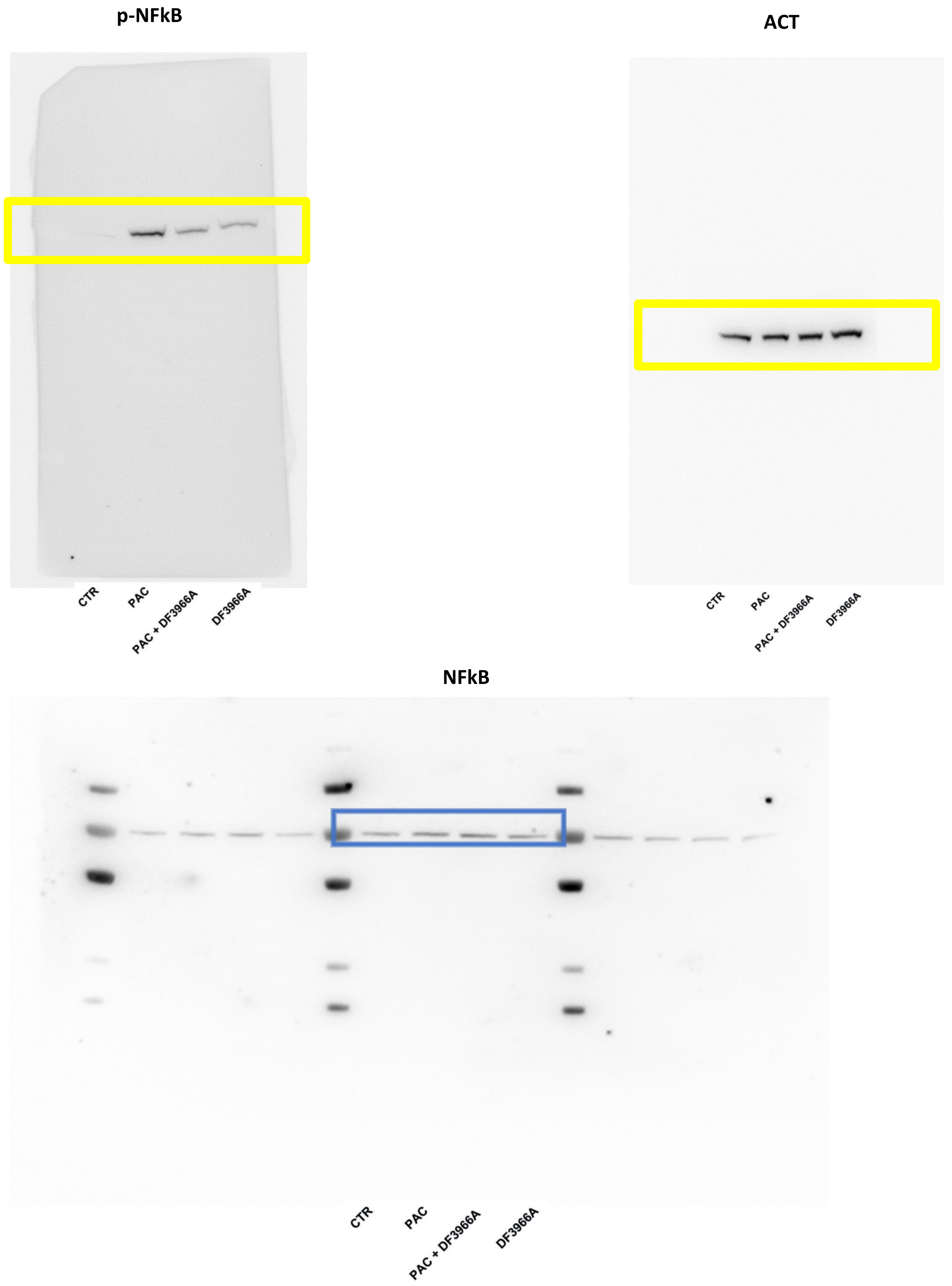

B

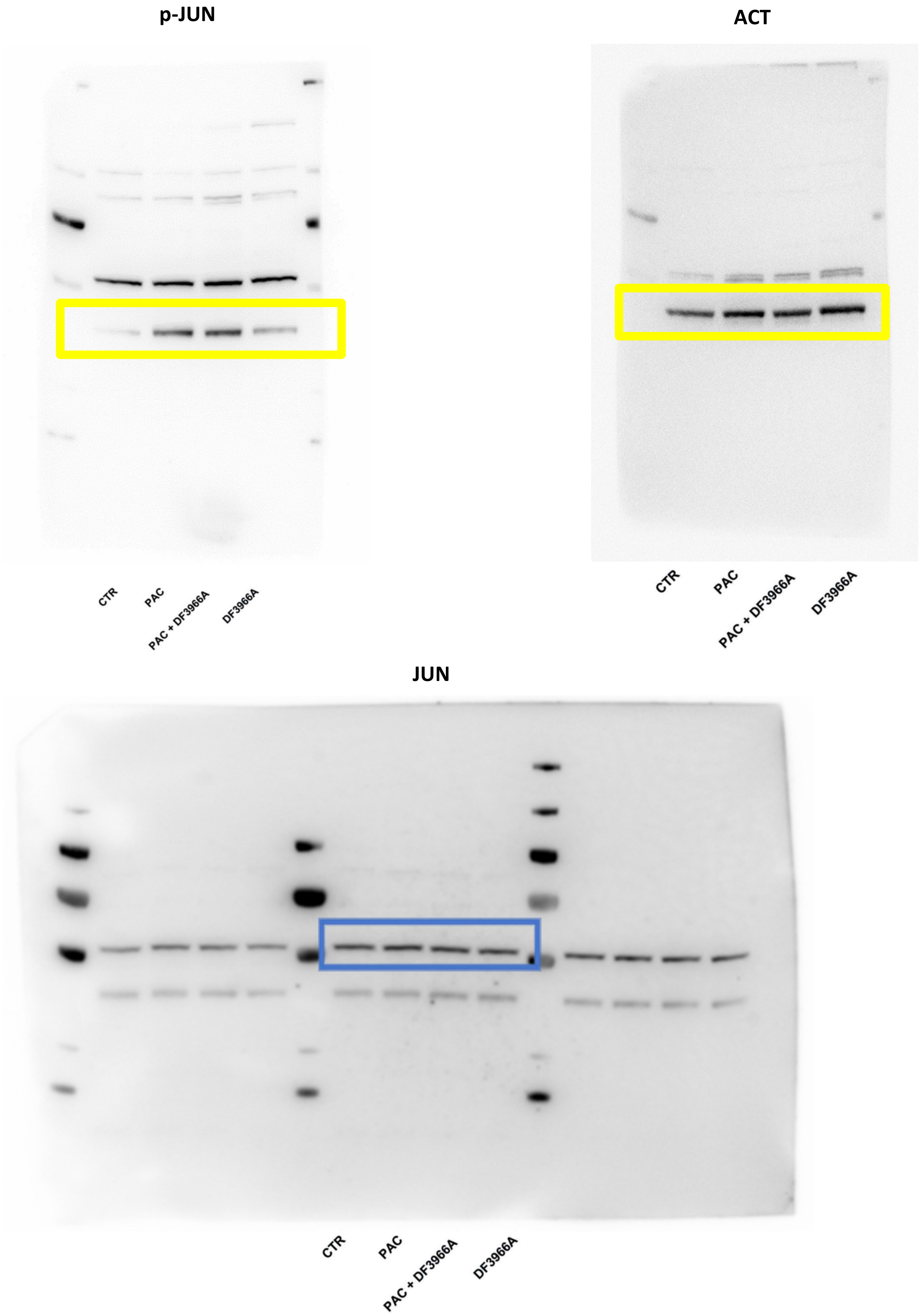
p-JUN
ACT
CTR
PAC
PAC + DF3966A
DF3966A
CTR
PAC
PAC + DF3966A
DF3966A
JUN
CTR
PAC
PAC + DF3966A
DF3966A

C

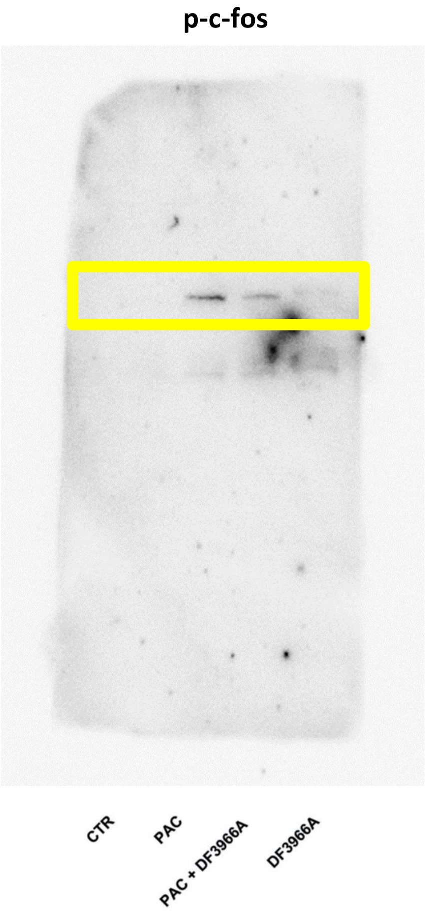
p-c-fos
CTR
PAC
PAC + DF3966A
DF3966A

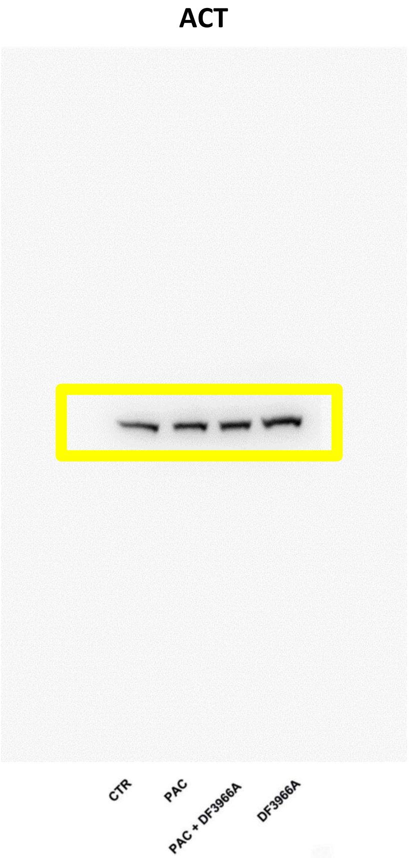
ACT
CTR
PAC
PAC + DF3966A
DF3966A

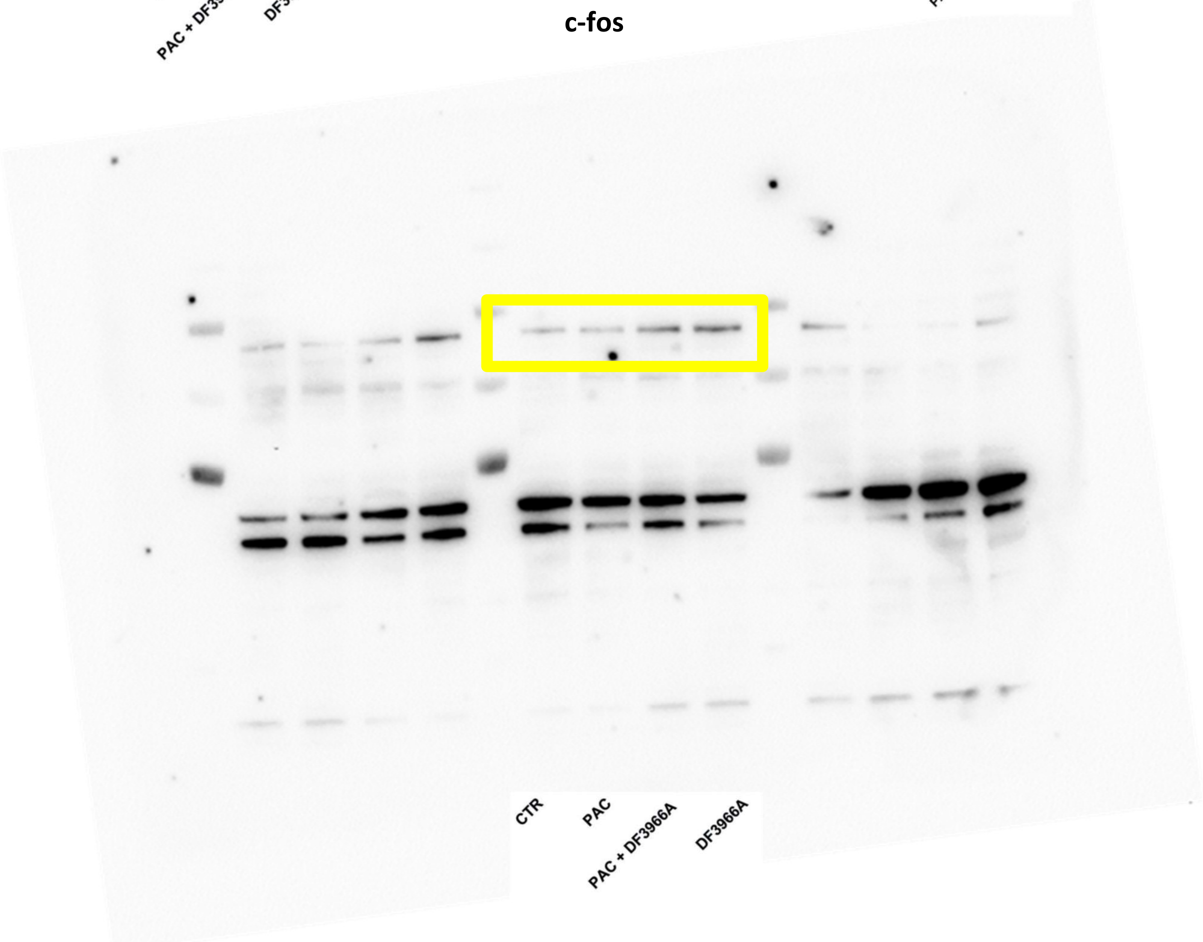
c-fos
CTR
PAC
PAC + DF3966A
DF3966A

D

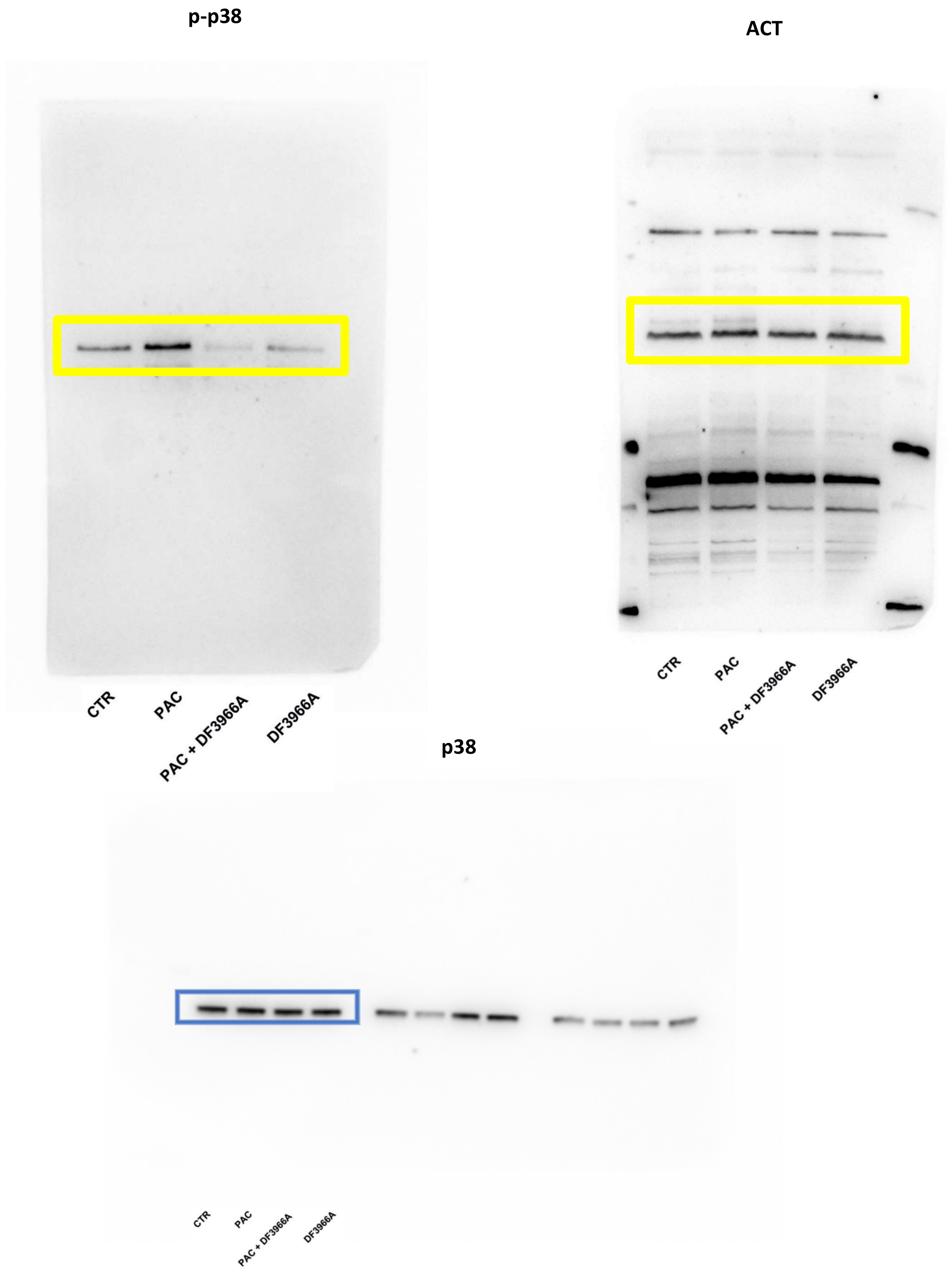
p-p38
ACT
CTR
PAC
PAC + DF3966A
DF3966A
CTR
PAC
PAC + DF3966A
DF3966A
p38
CTR
PAC
PAC + DF3966A
DF3966A

E

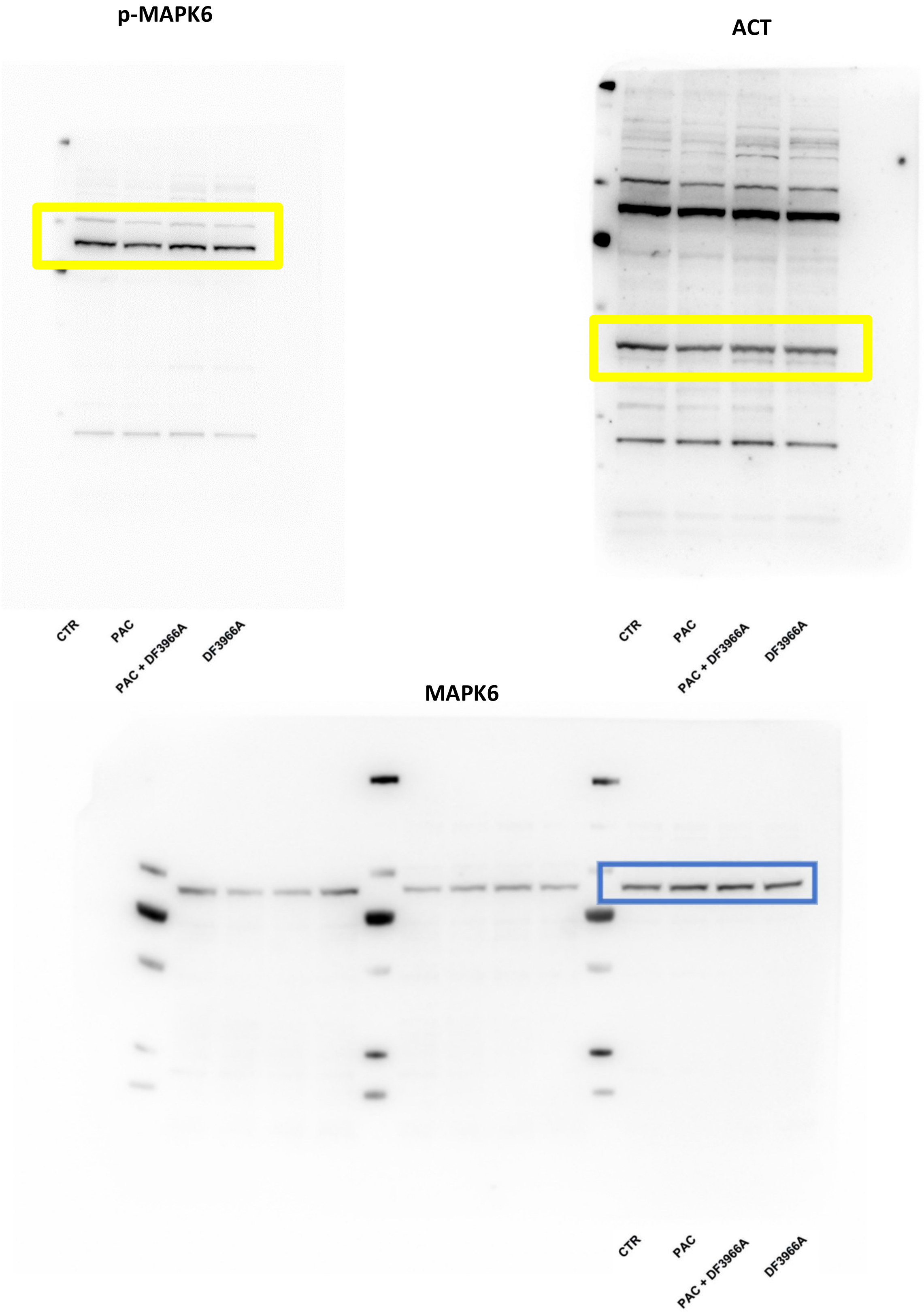
p-MAPK6
ACT
CTR
PAC
PAC + DF3966A
DF3966A
CTR
PAC
PAC + DF3966A
DF3966A
MAPK6
CTR
PAC
PAC + DF3966A
DF3966A

Full and uncropped western blot for FIGURE 4

A

p-Cortactin

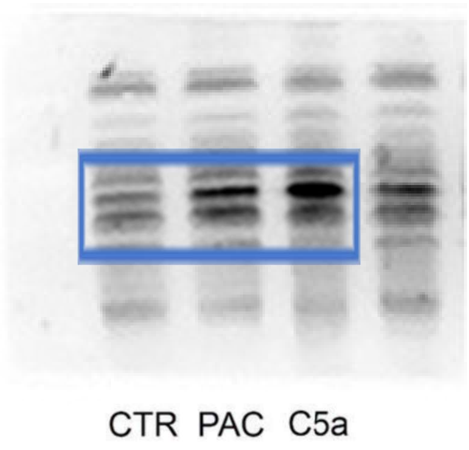

Cortactin

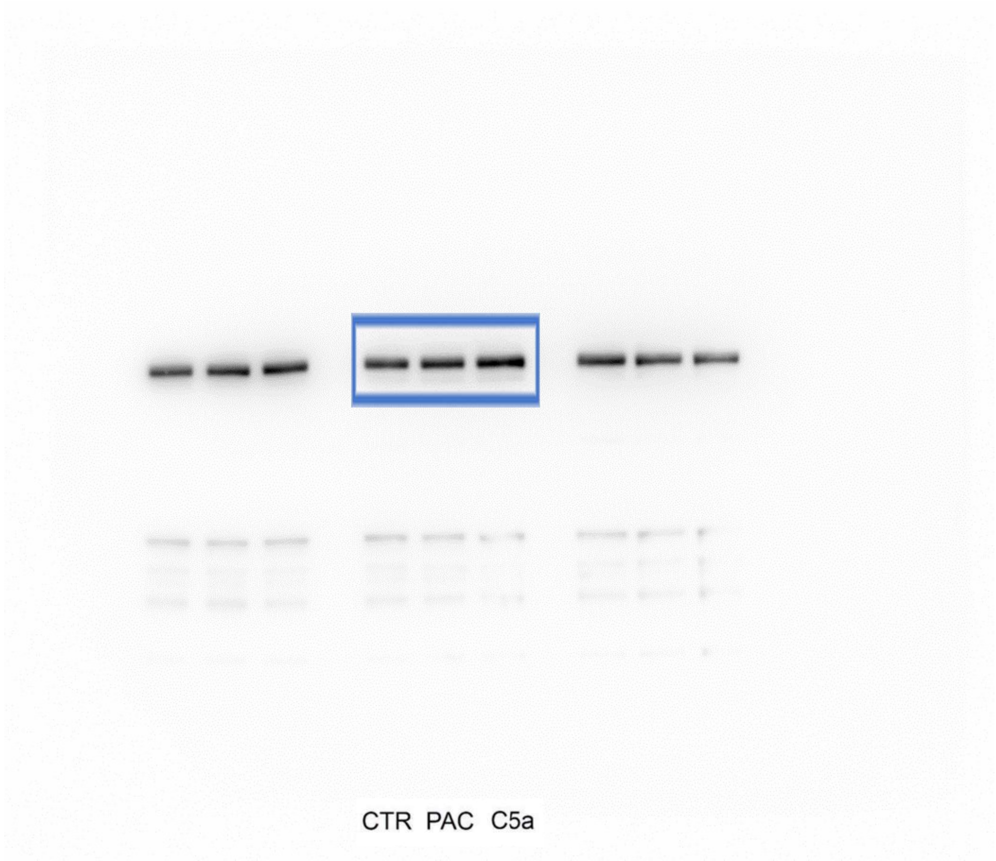

Actin

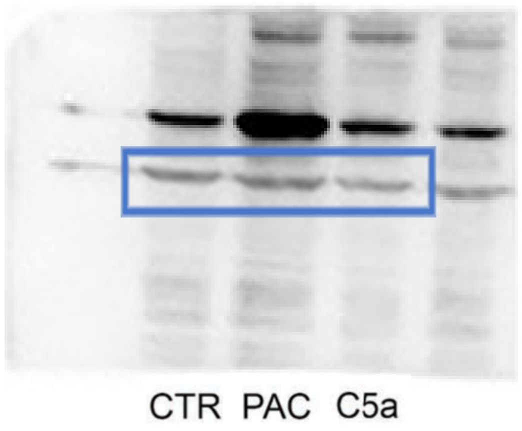

B

p-Fak

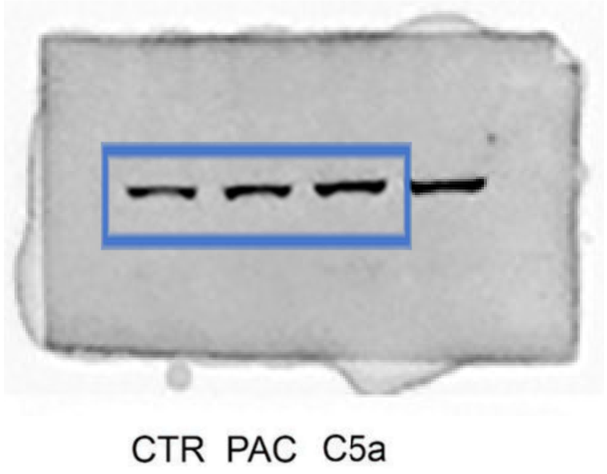

Fak

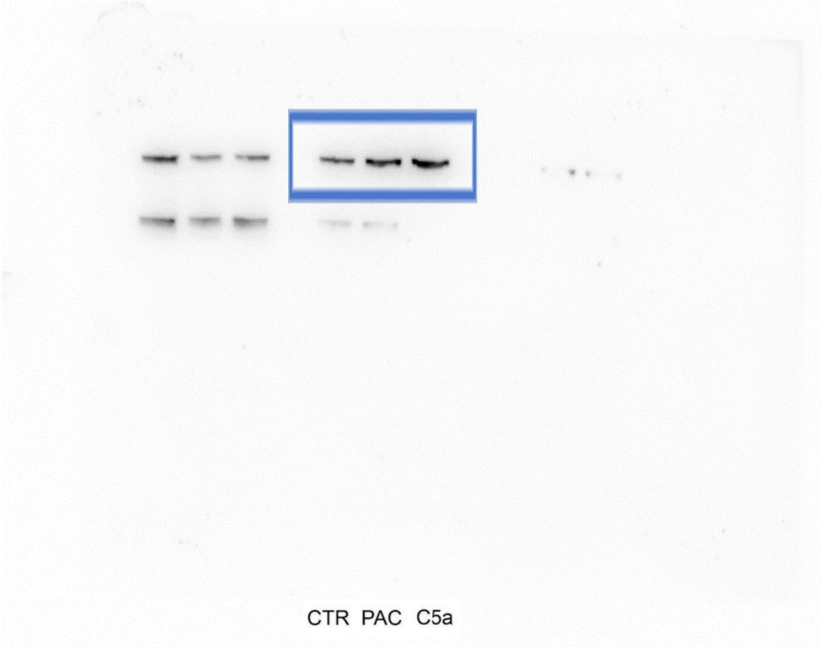

Actin

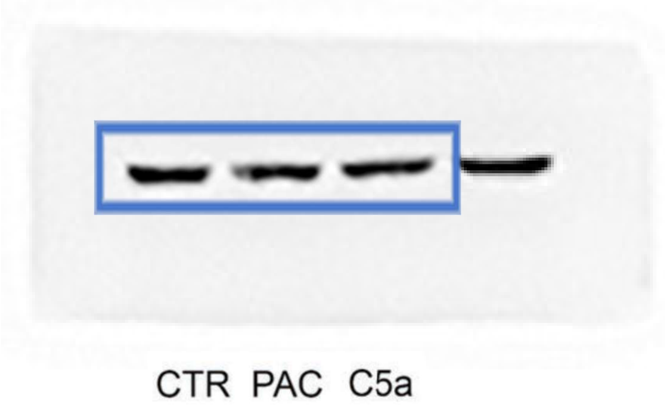

C

p-Jak

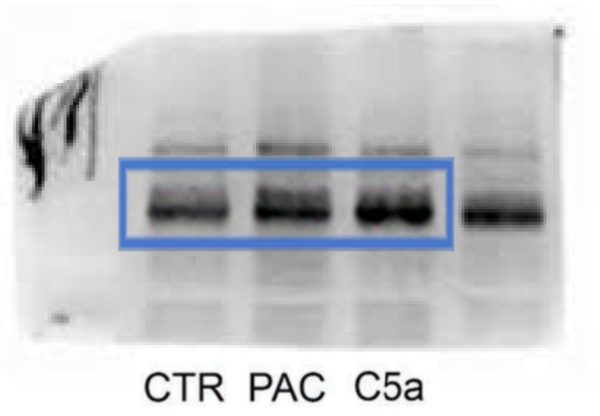

Jak

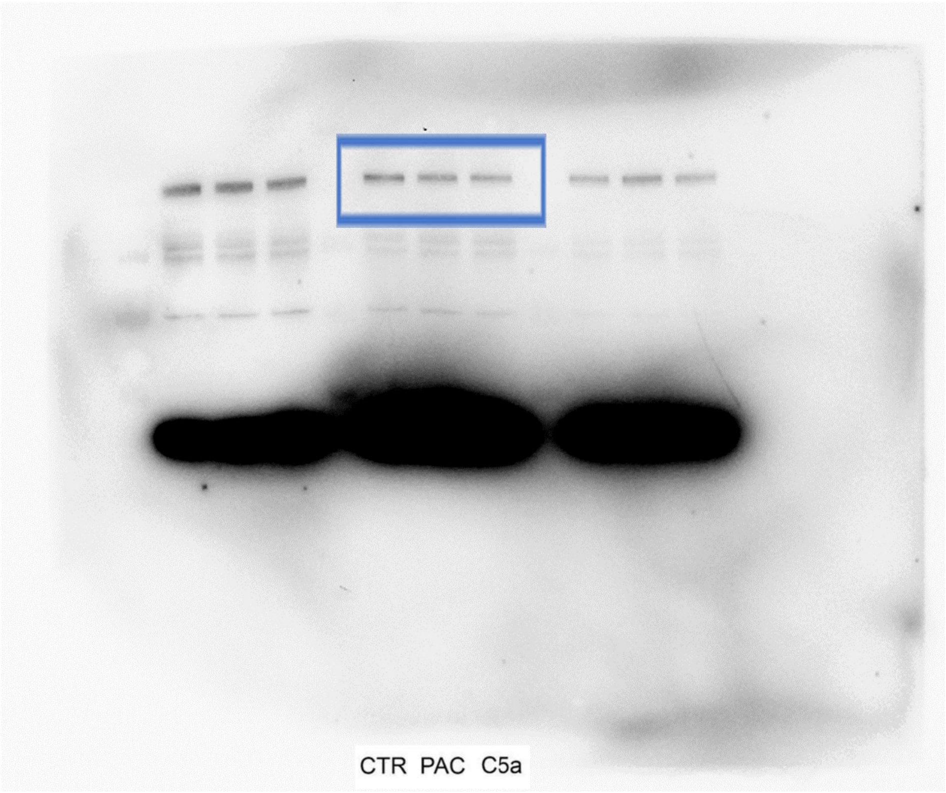

Actin

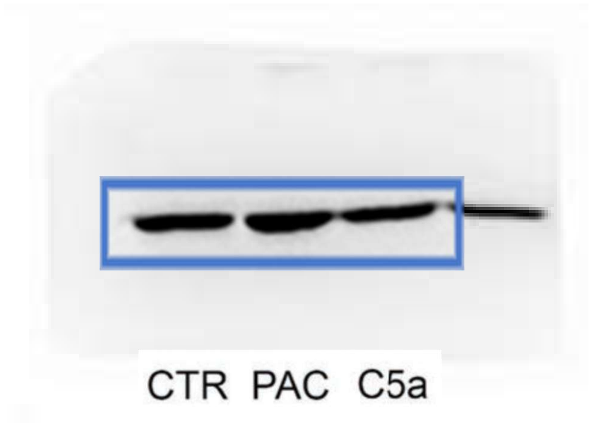

D

p-Stat3

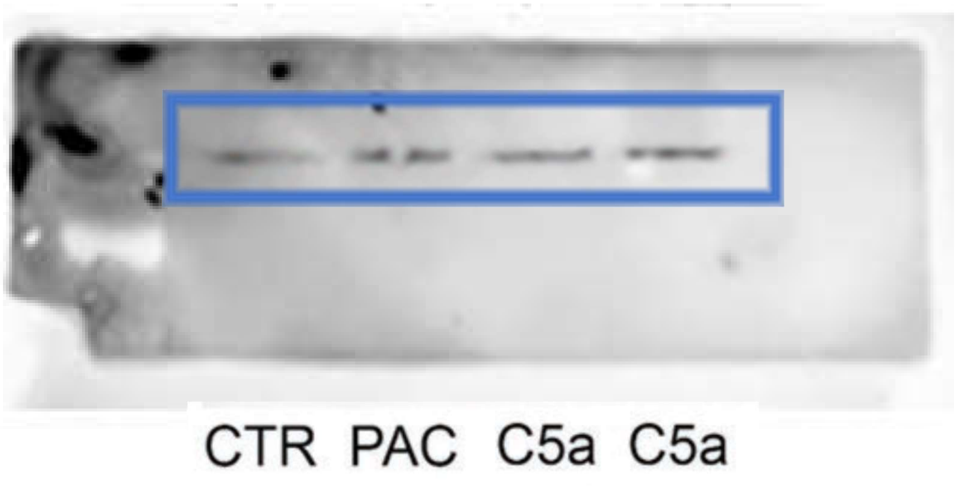

Stat3

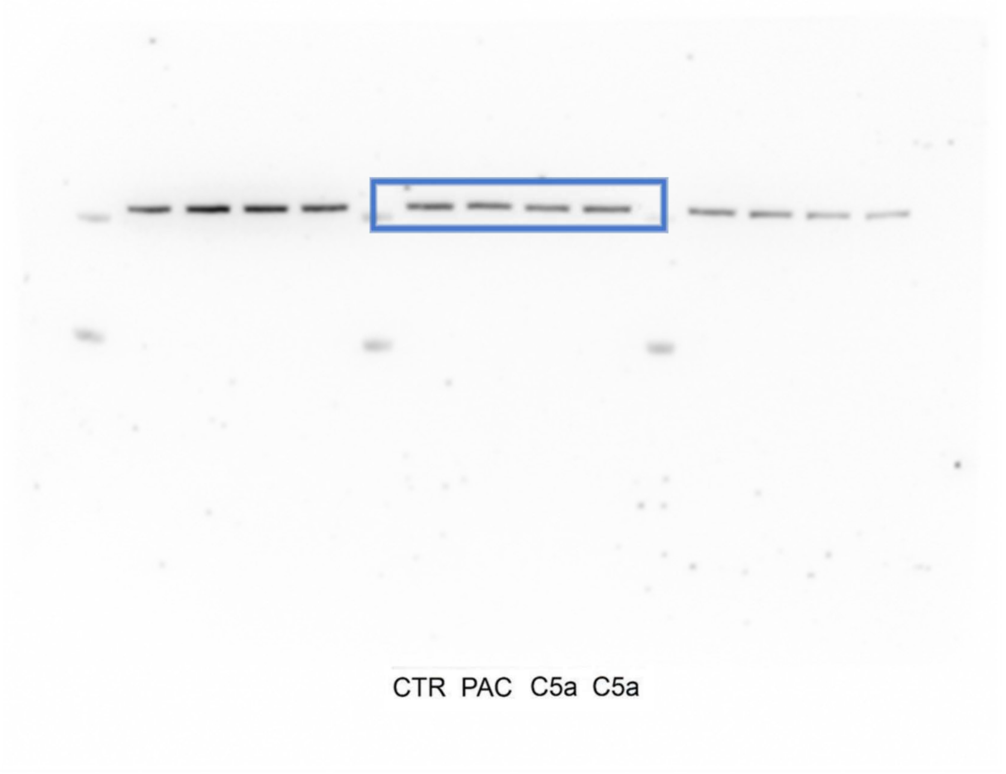

Actin

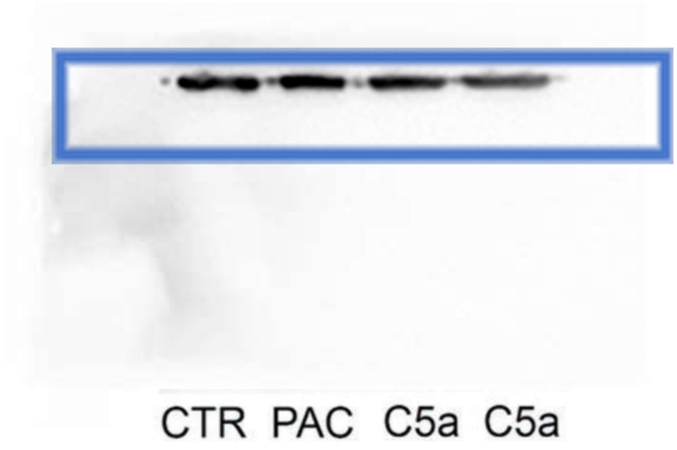

E

p-Fak

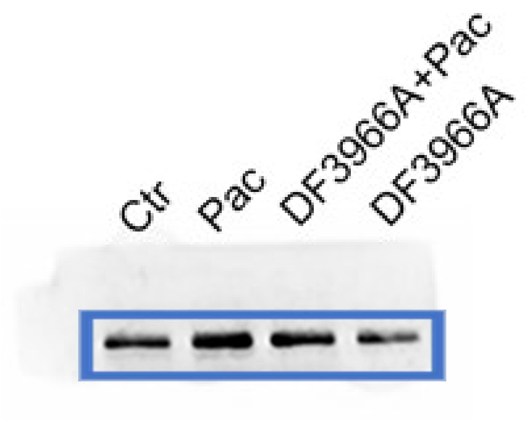

Fak

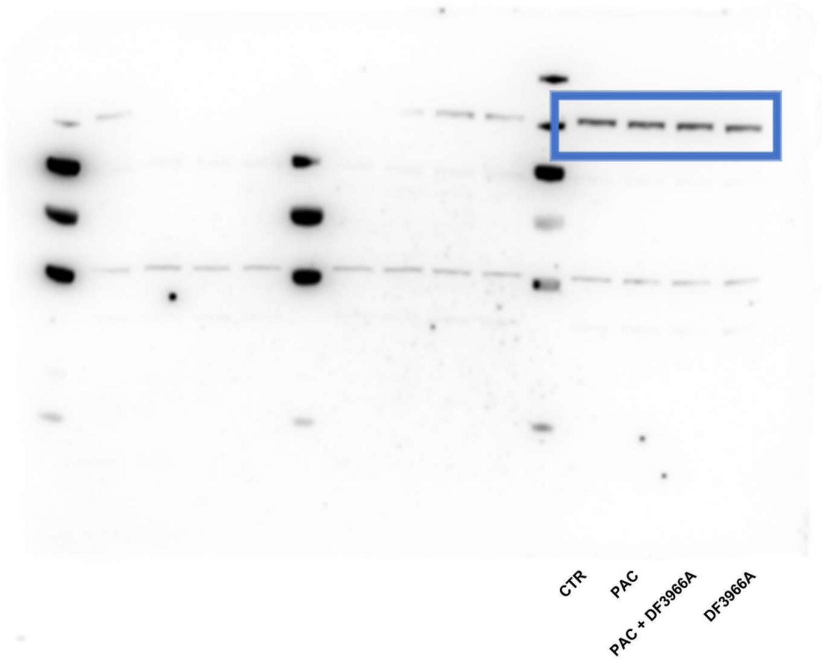

p-Cortactin

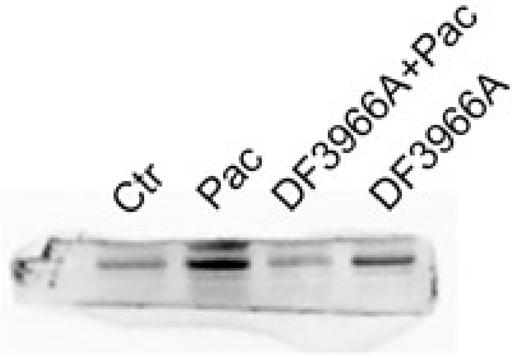

Cortactin

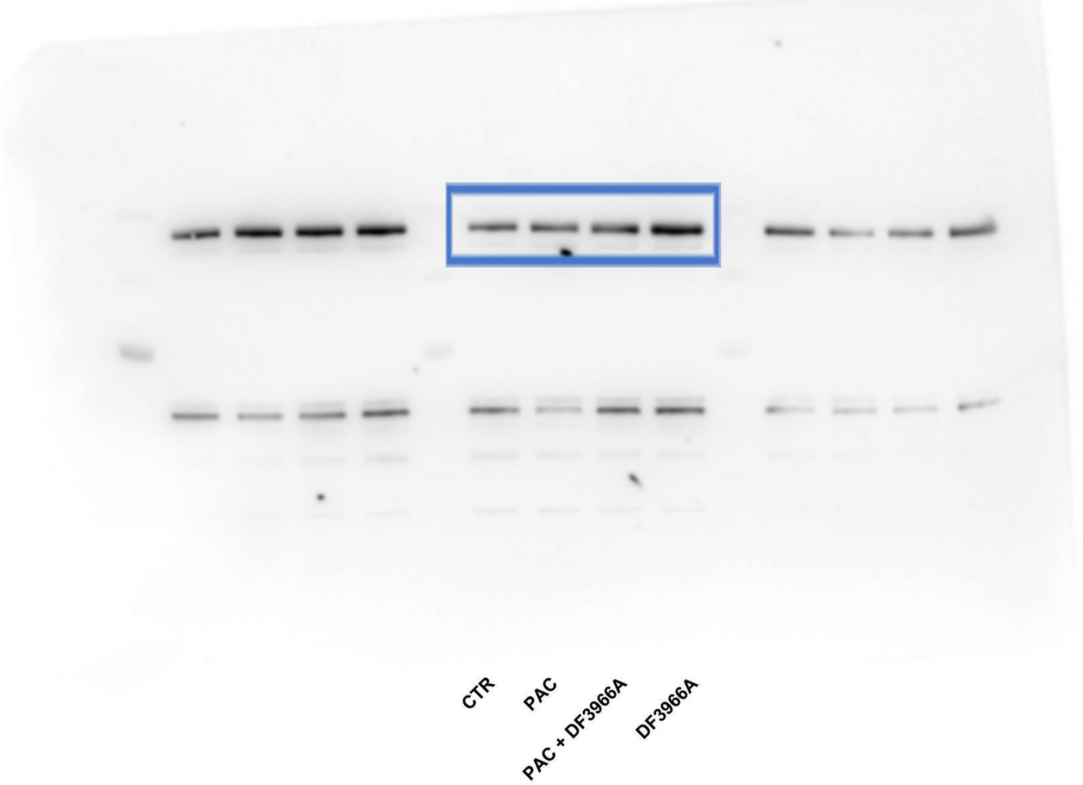

Actin

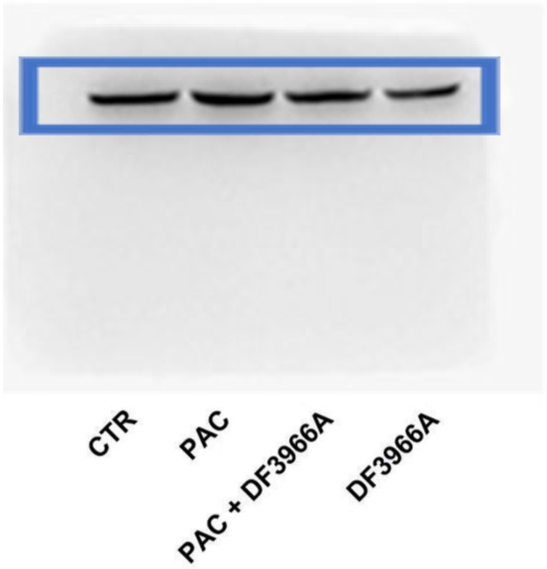

F

p-Jak

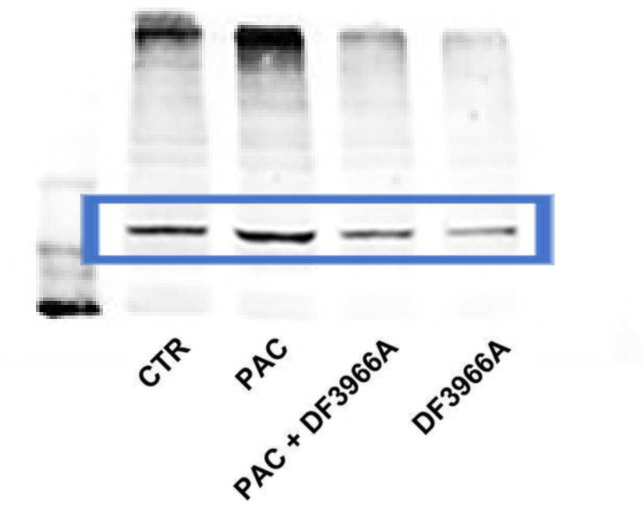

Jak

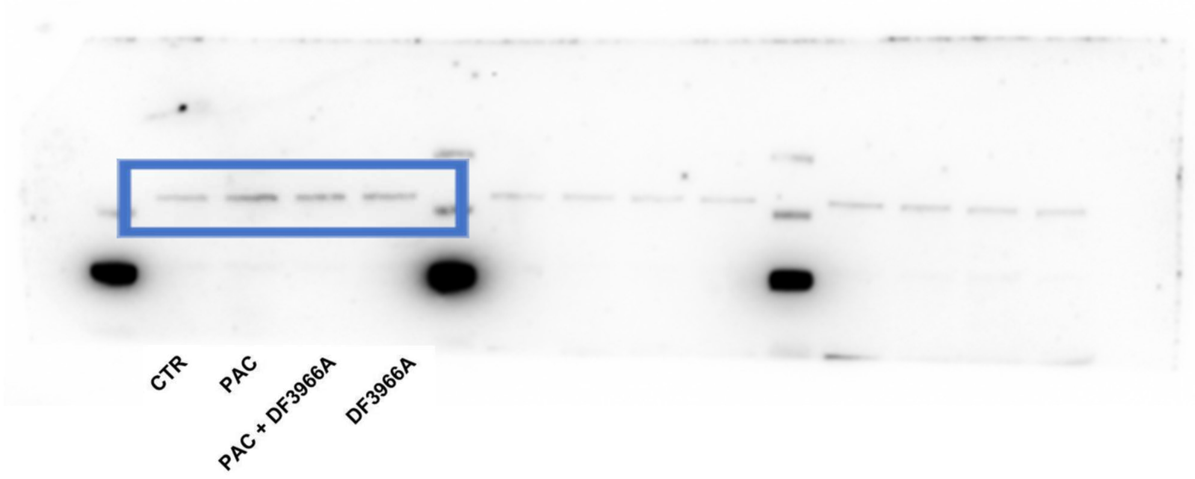

Actin

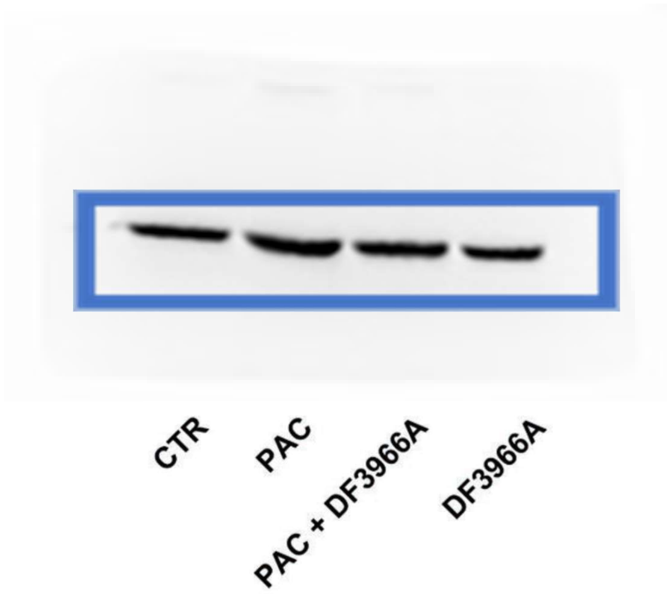

p-Stat3

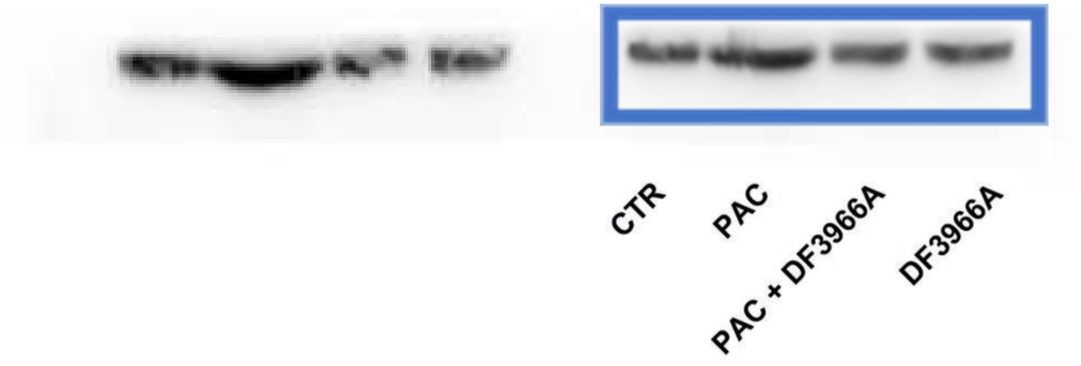

Stat3

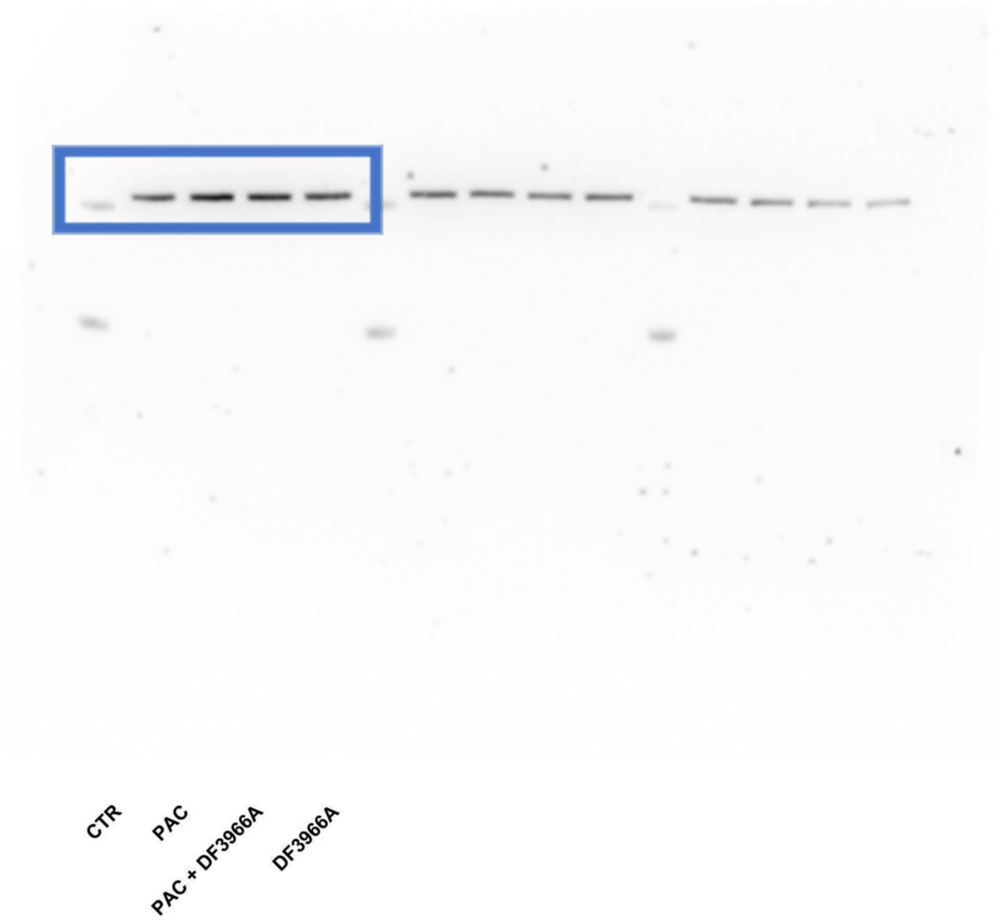

Actin

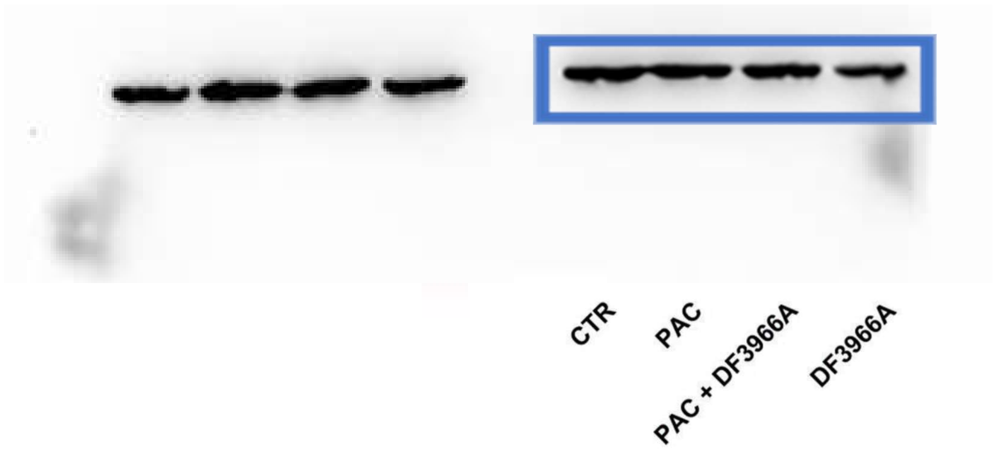

Full and uncropped western blot for <u>FIGURE S3</u>

A

p-NFkB

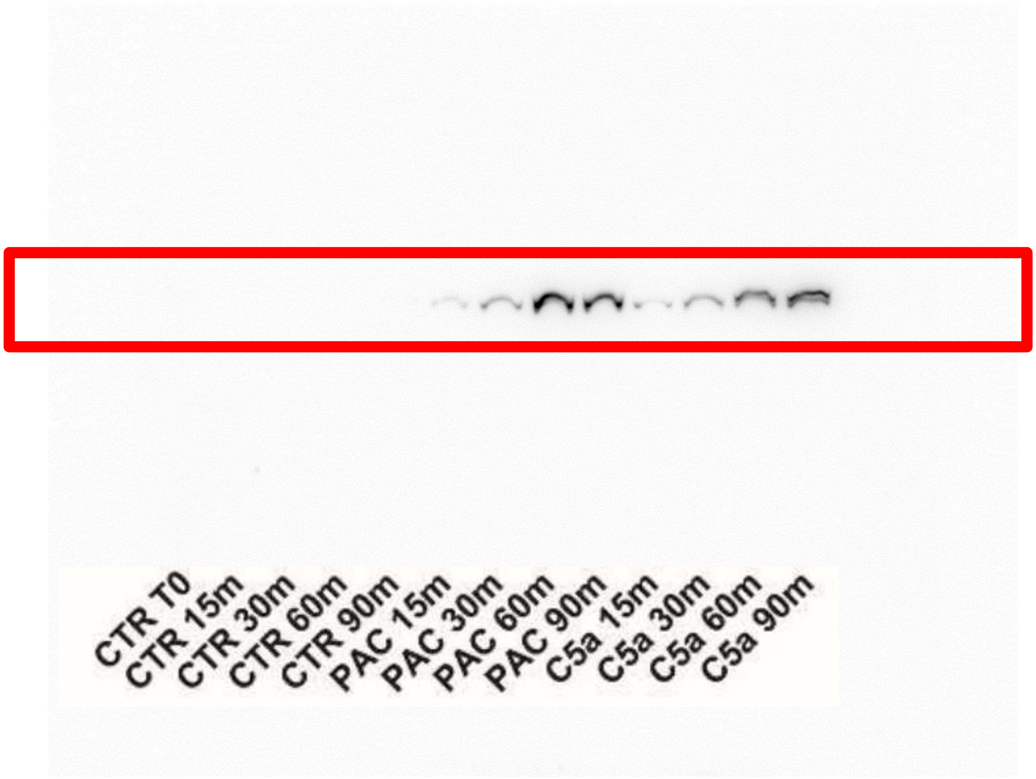

Actin

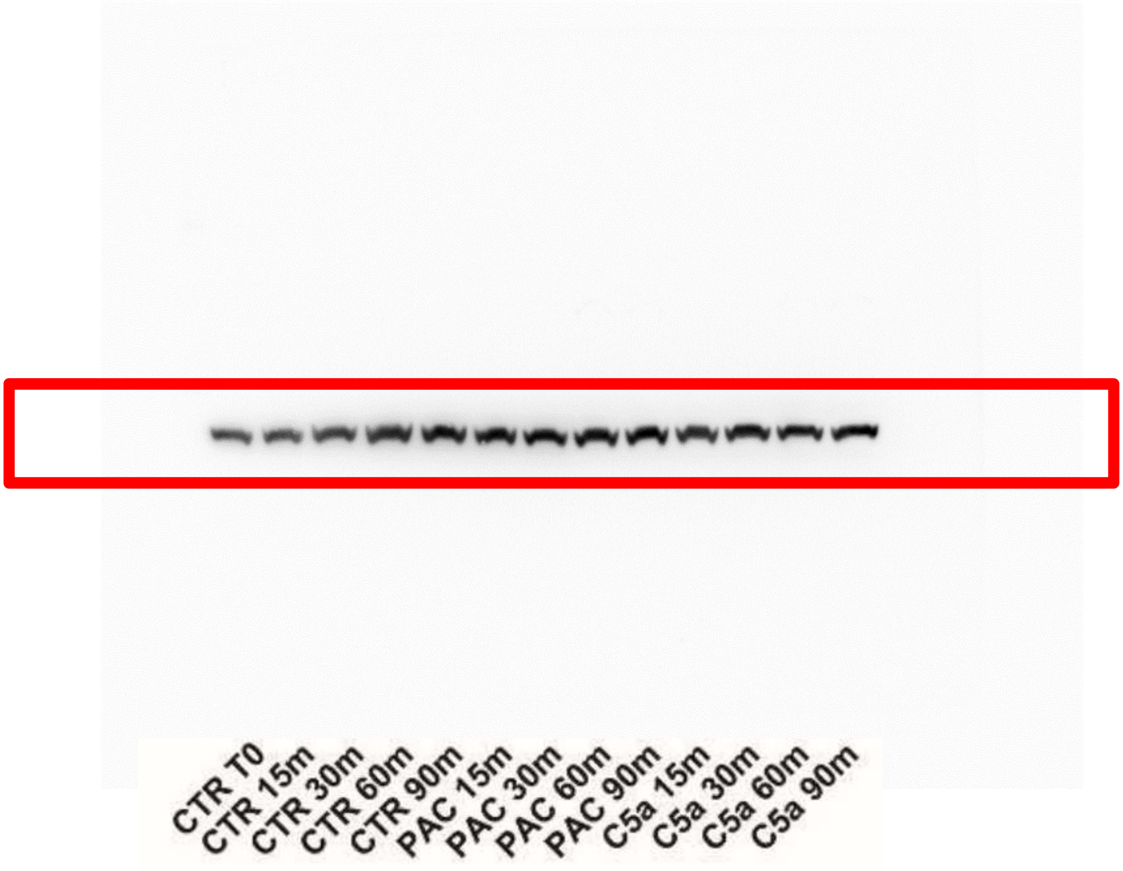

p-Jun

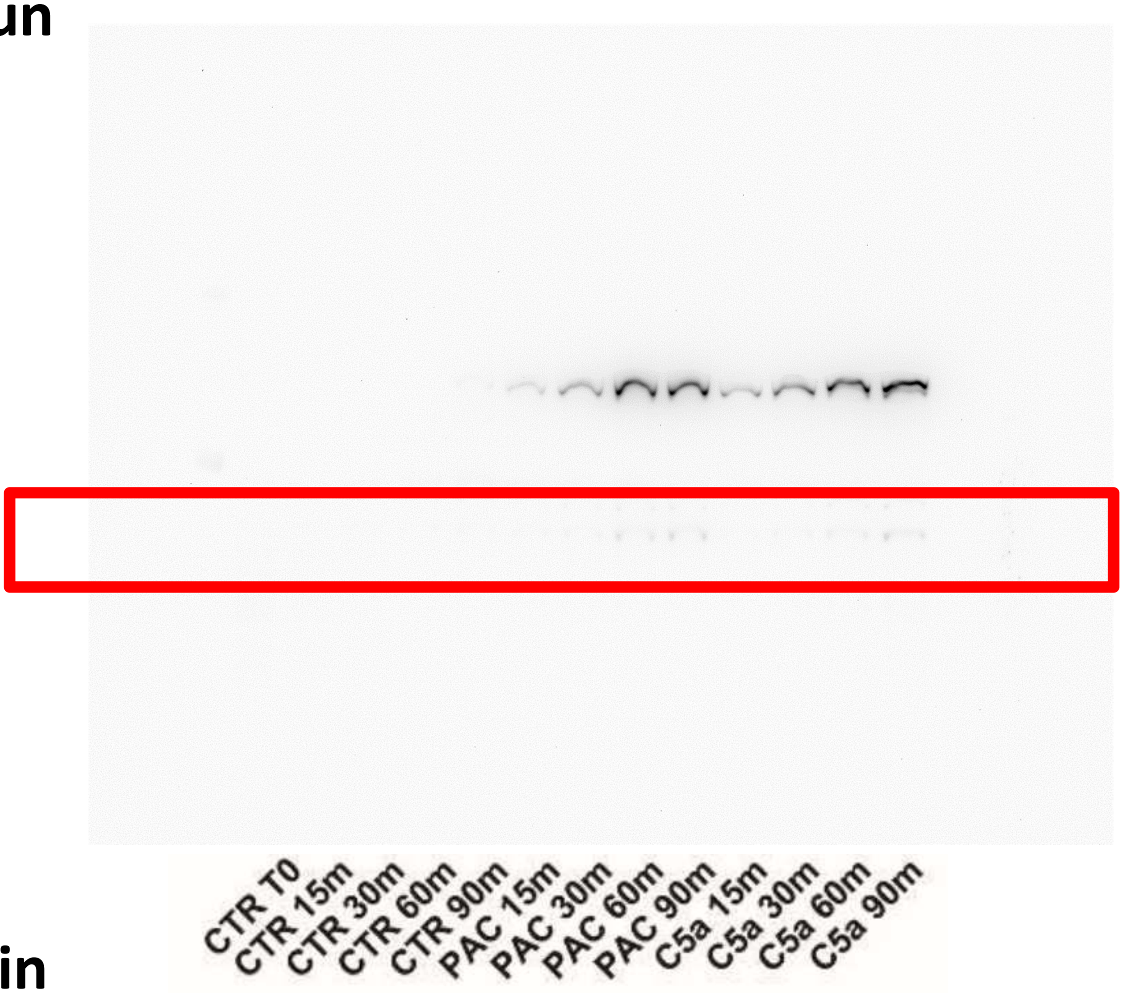
CTR T0
CTR 15m
CTR 30m
CTR 60m
CTR 90m
PAC 15m
PAC 30m
PAC 60m
PAC 90m
C5a 15m
C5a 30m
C5a 60m
C5a 90m

Actin

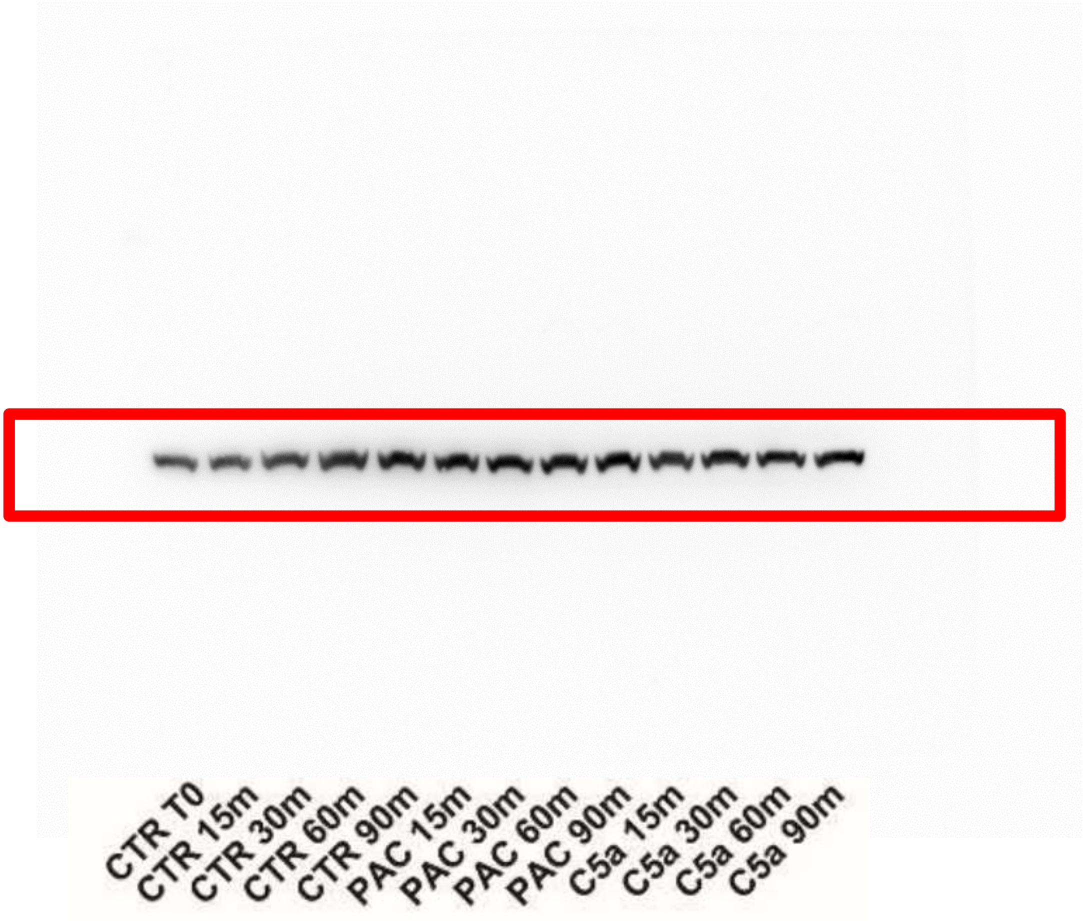
CTR T0
CTR 15m
CTR 30m
CTR 60m
CTR 90m
PAC 15m
PAC 30m
PAC 60m
PAC 90m
C5a 15m
C5a 30m
C5a 60m
C5a 90m

p-C-FOS

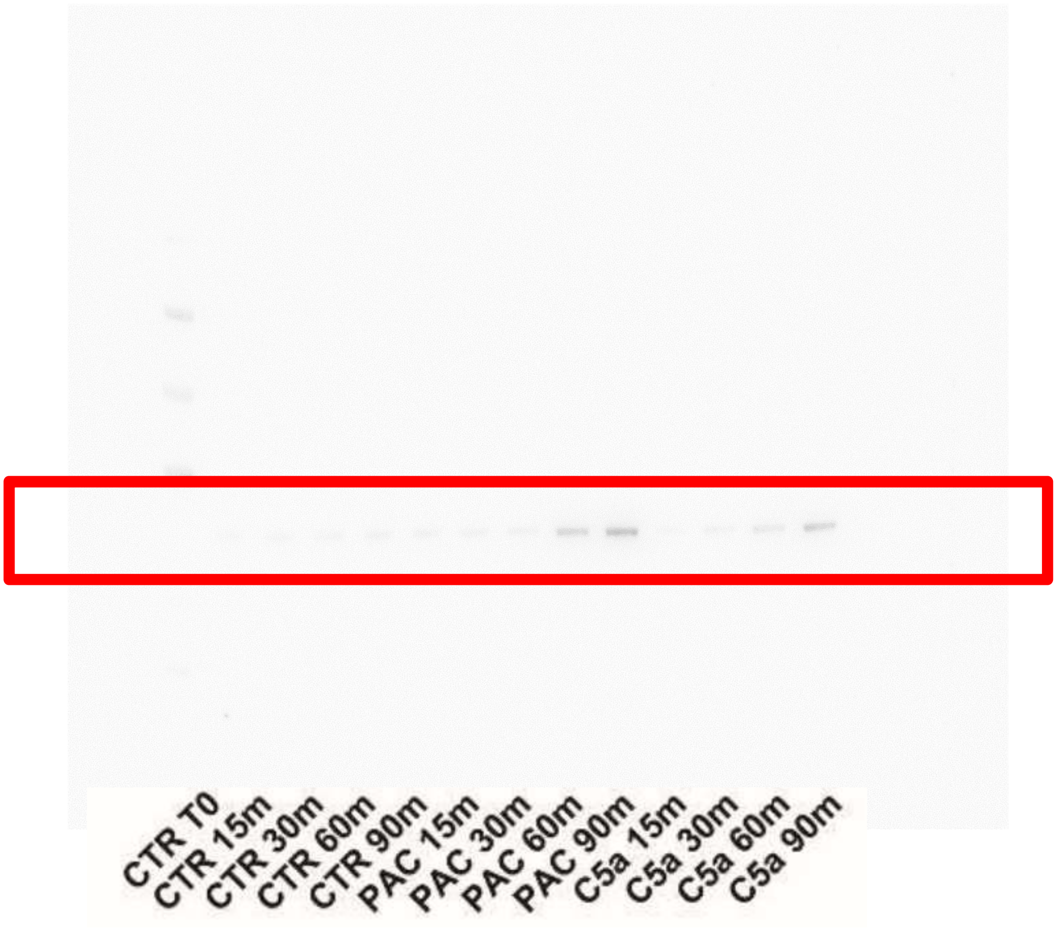
CTR T0
CTR 15m
CTR 30m
CTR 60m
CTR 90m
PAC 15m
PAC 30m
PAC 60m
PAC 90m
C5a 15m
C5a 30m
C5a 60m
C5a 90m

Actin

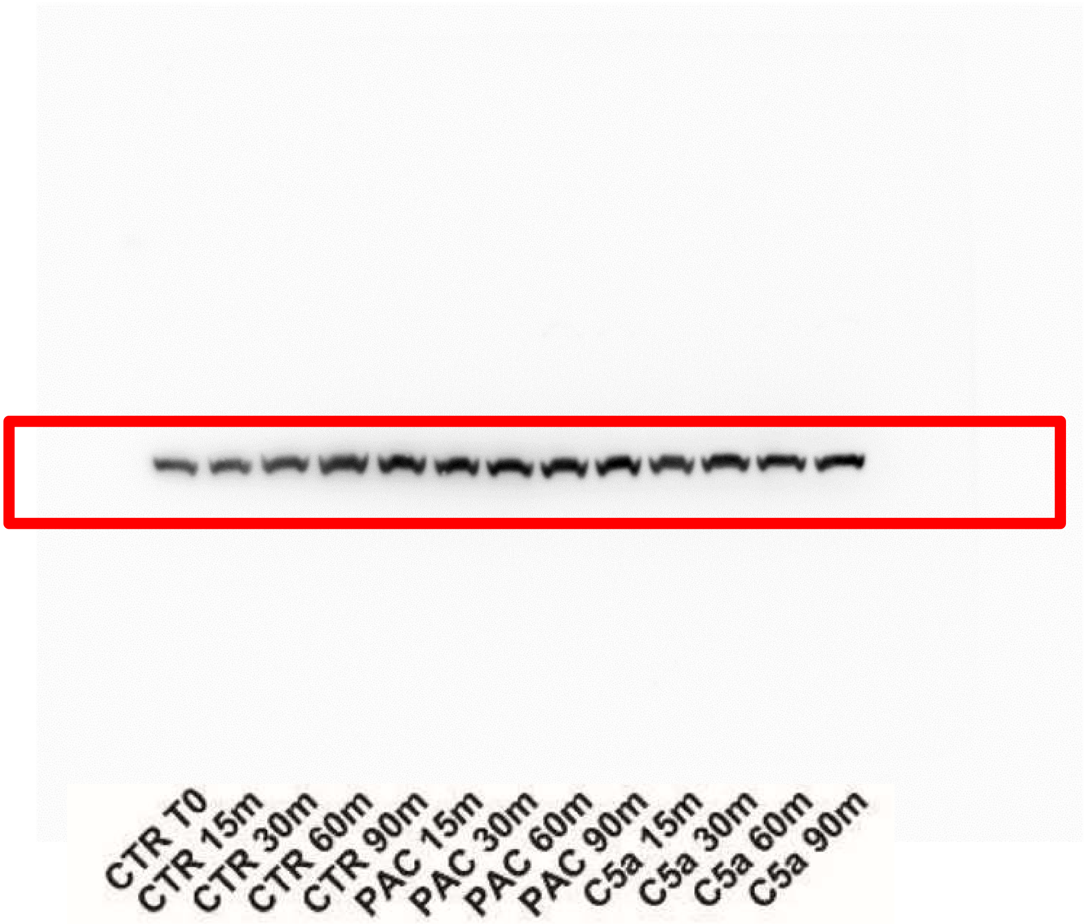
CTR T0
CTR 15m
CTR 30m
CTR 60m
CTR 90m
PAC 15m
PAC 30m
PAC 60m
PAC 90m
C5a 15m
C5a 30m
C5a 60m
C5a 90m

p-P38

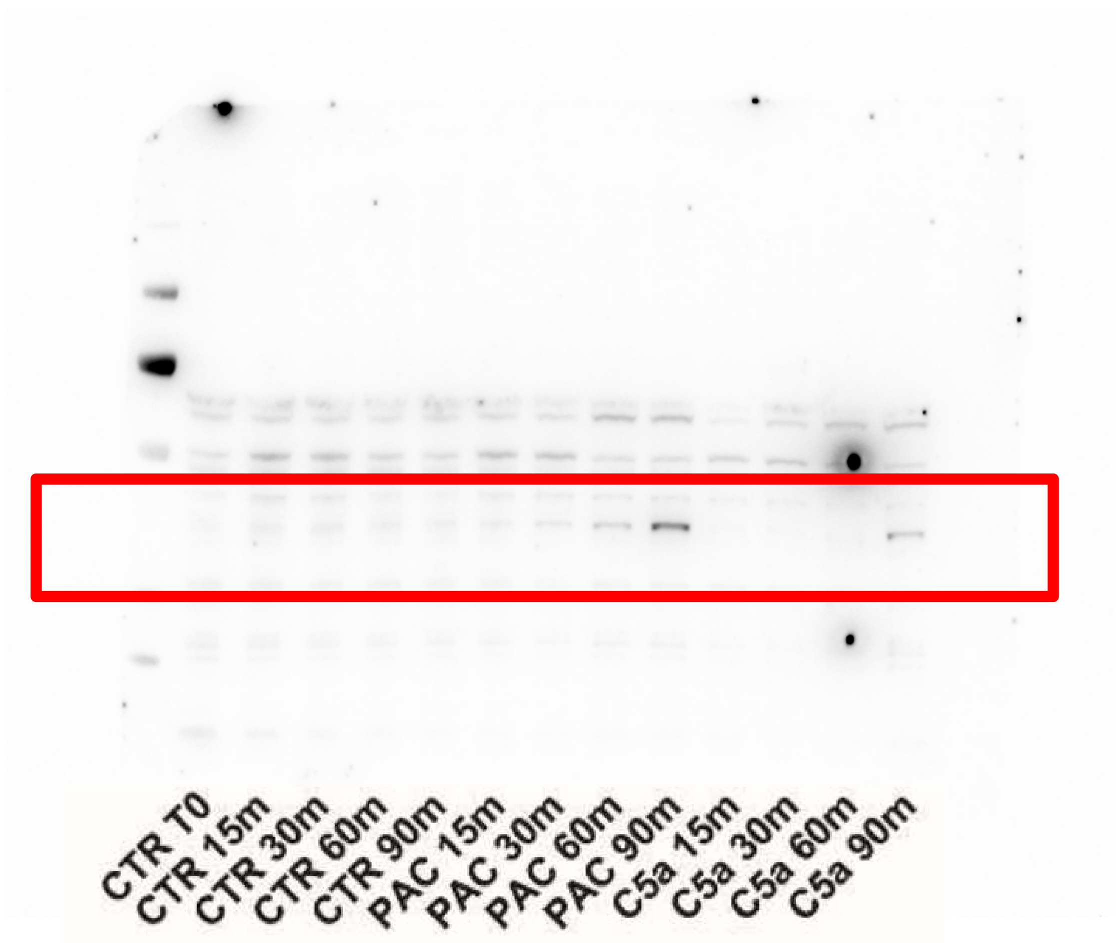
CTR T0
CTR 15m
CTR 30m
CTR 60m
CTR 90m
PAC 15m
PAC 30m
PAC 60m
PAC 90m
C5a 15m
C5a 30m
C5a 60m
C5a 90m

Actin

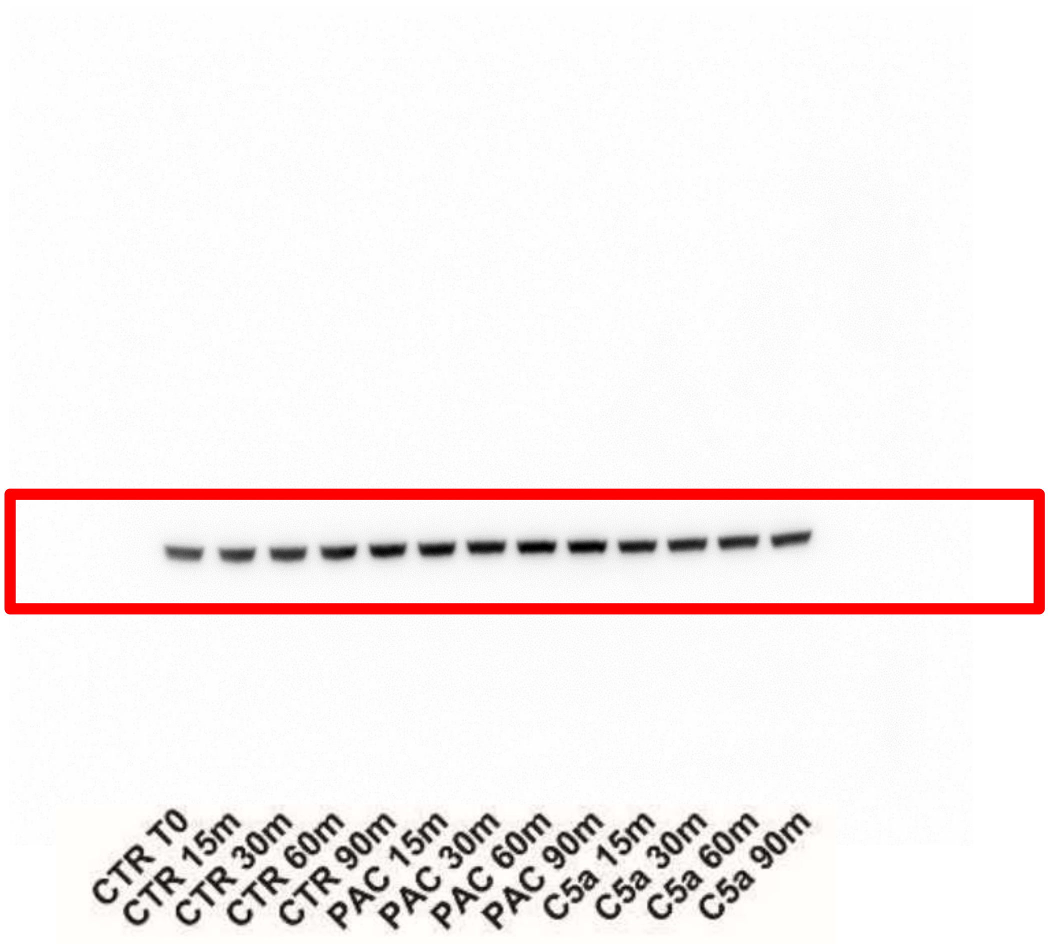
CTR T0
CTR 15m
CTR 30m
CTR 60m
CTR 90m
PAC 15m
PAC 30m
PAC 60m
PAC 90m
C5a 15m
C5a 30m
C5a 60m
C5a 90m

B

p-Fak

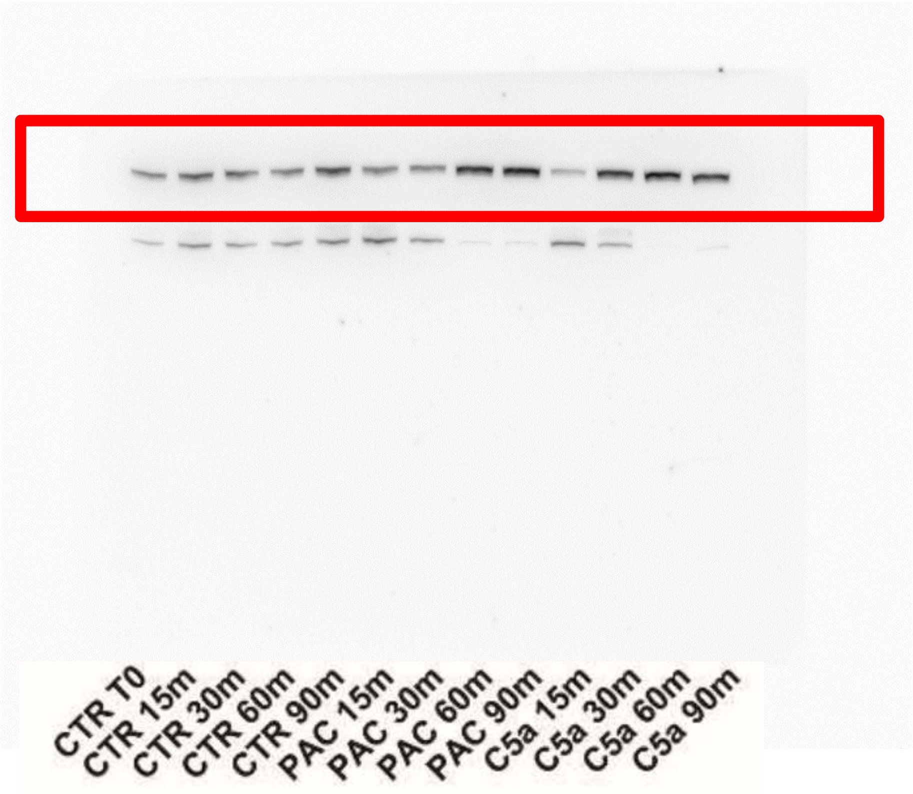
CTR T0
CTR 15m
CTR 30m
CTR 60m
CTR 90m
PAC 15m
PAC 30m
PAC 60m
PAC 90m
C5a 15m
C5a 30m
C5a 60m
C5a 90m

Actin

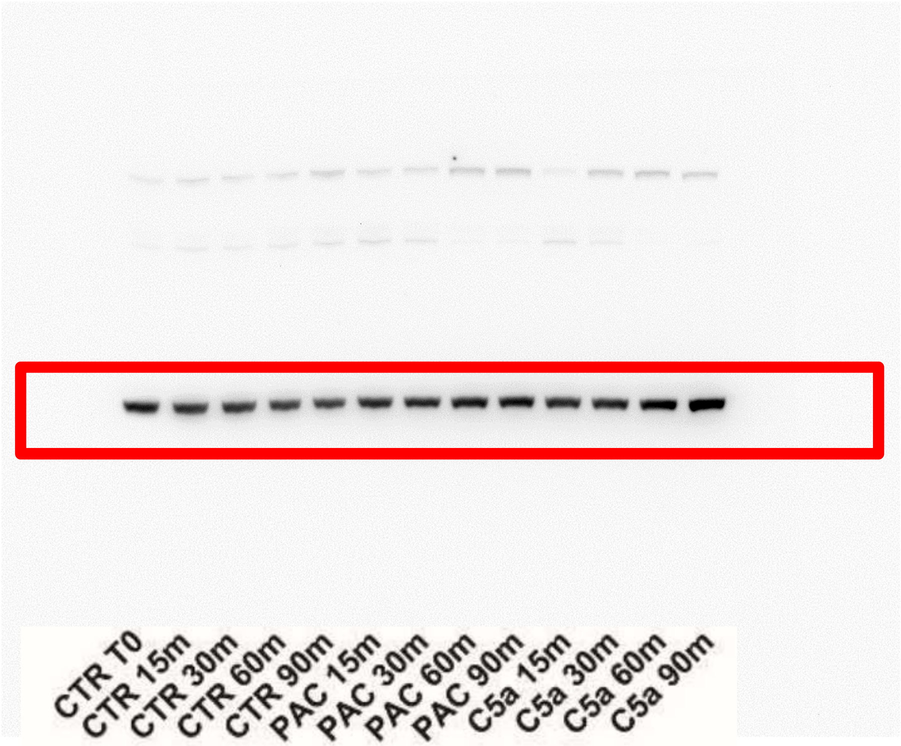
CTR T0
CTR 15m
CTR 30m
CTR 60m
CTR 90m
PAC 15m
PAC 30m
PAC 60m
PAC 90m
C5a 15m
C5a 30m
C5a 60m
C5a 90m

p-CORT

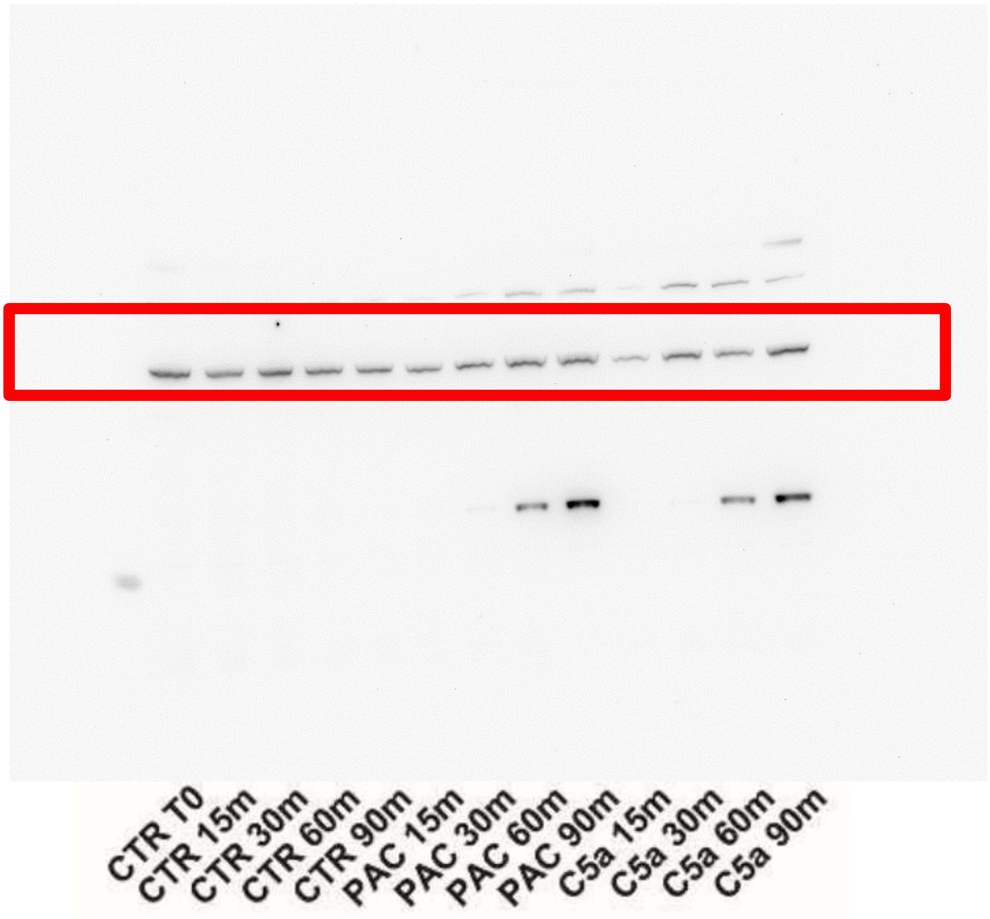
CTR T0
CTR 15m
CTR 30m
CTR 60m
CTR 90m
PAC 15m
PAC 30m
PAC 60m
PAC 90m
C5a 15m
C5a 30m
C5a 60m
C5a 90m

Actin

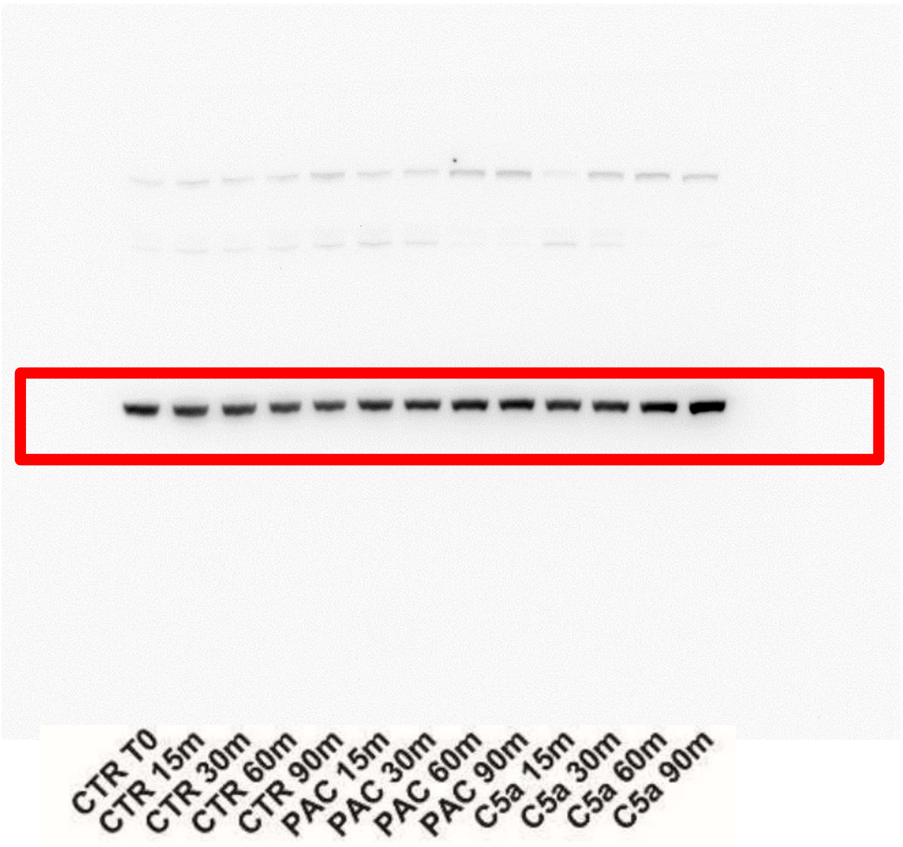
CTR T0
CTR 15m
CTR 30m
CTR 60m
CTR 90m
PAC 15m
PAC 30m
PAC 60m
PAC 90m
C5a 15m
C5a 30m
C5a 60m
C5a 90m

p-JAK

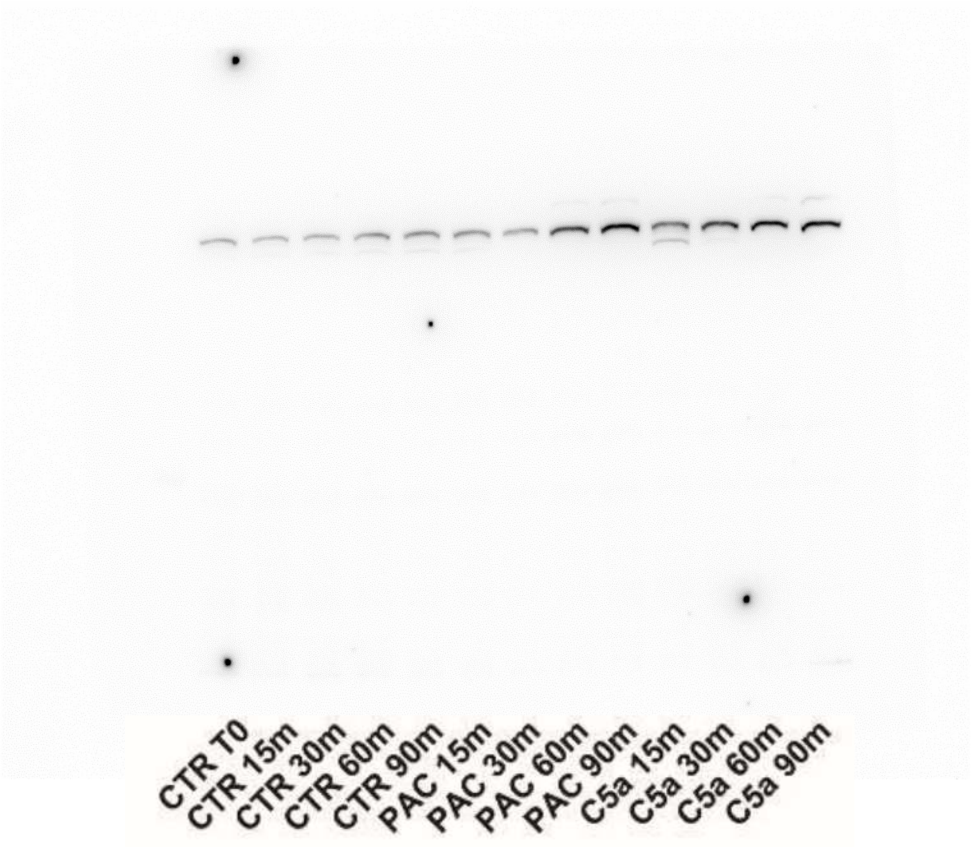
CTR T0
CTR 15m
CTR 30m
CTR 60m
CTR 90m
PAC 15m
PAC 30m
PAC 60m
PAC 90m
C5a 15m
C5a 30m
C5a 60m
C5a 90m

Actin

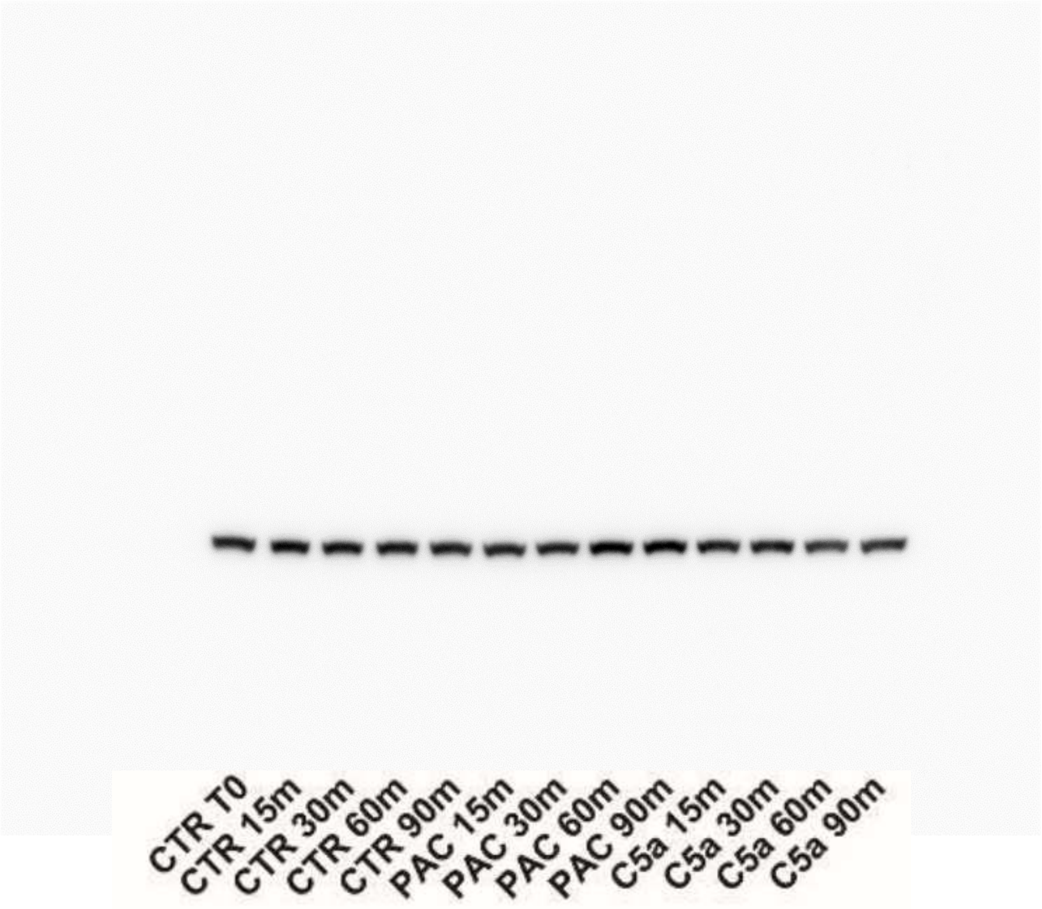
CTR T0
CTR 15m
CTR 30m
CTR 60m
CTR 90m
PAC 15m
PAC 30m
PAC 60m
PAC 90m
C5a 15m
C5a 30m
C5a 60m
C5a 90m

p-MAPK

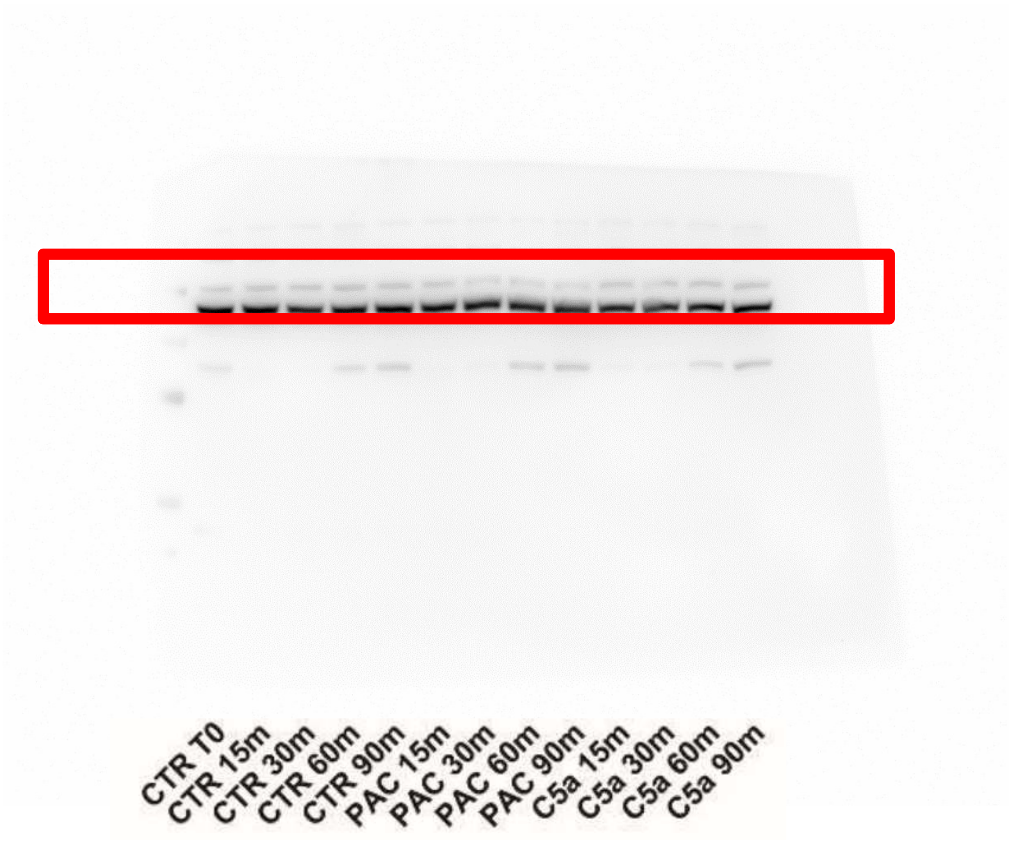
CTR T0
CTR 15m
CTR 30m
CTR 60m
CTR 90m
PAC 15m
PAC 30m
PAC 60m
PAC 90m
C5a 15m
C5a 30m
C5a 60m
C5a 90m

Actin

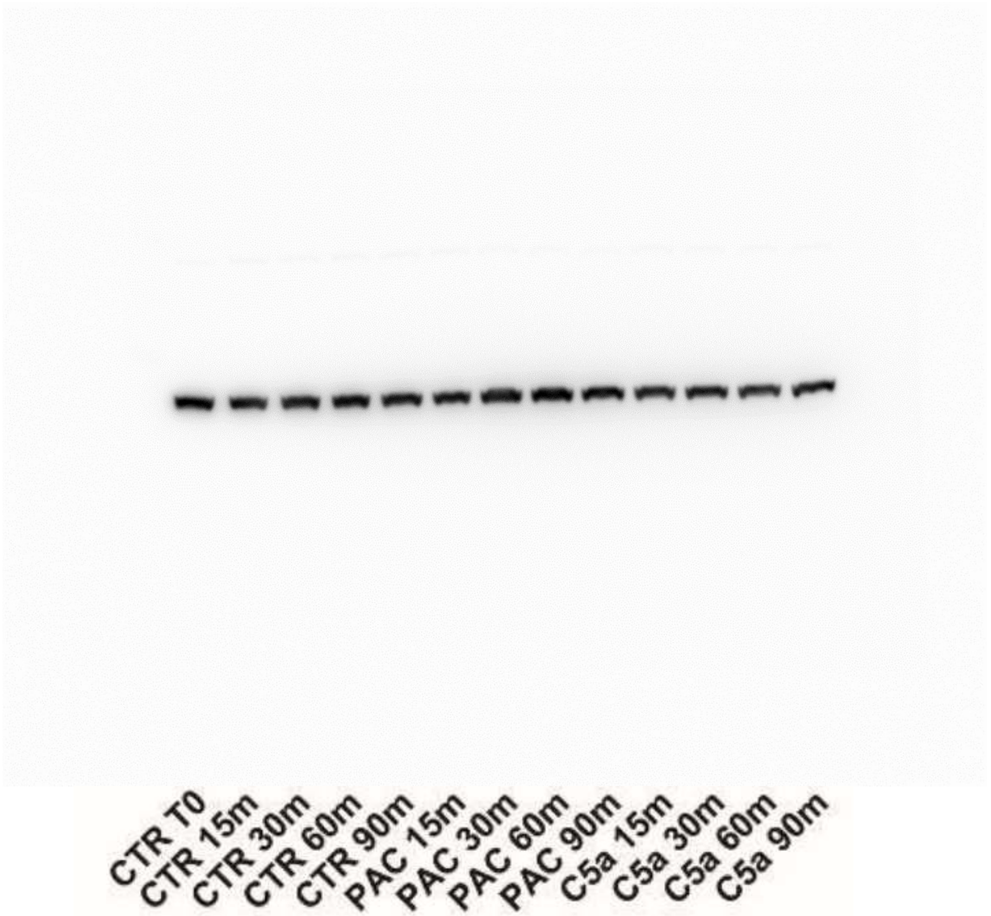
CTR T0
CTR 15m
CTR 30m
CTR 60m
CTR 90m
PAC 15m
PAC 30m
PAC 60m
PAC 90m
C5a 15m
C5a 30m
C5a 60m
C5a 90m

Full and uncropped western blot for <u>Figure S4 A</u>

C5aR1

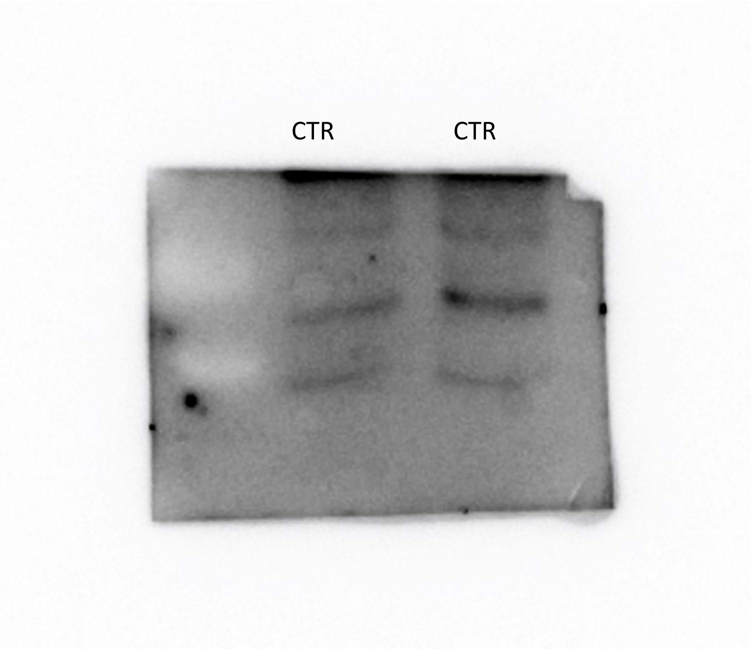

Actin

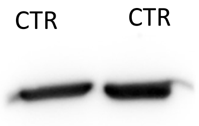

Full and uncropped western blot for <u>Figure S5</u>

p-Fak

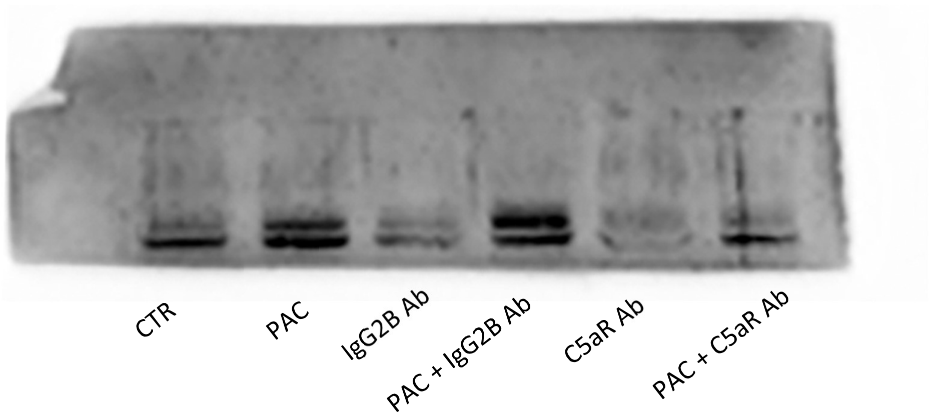

Actin

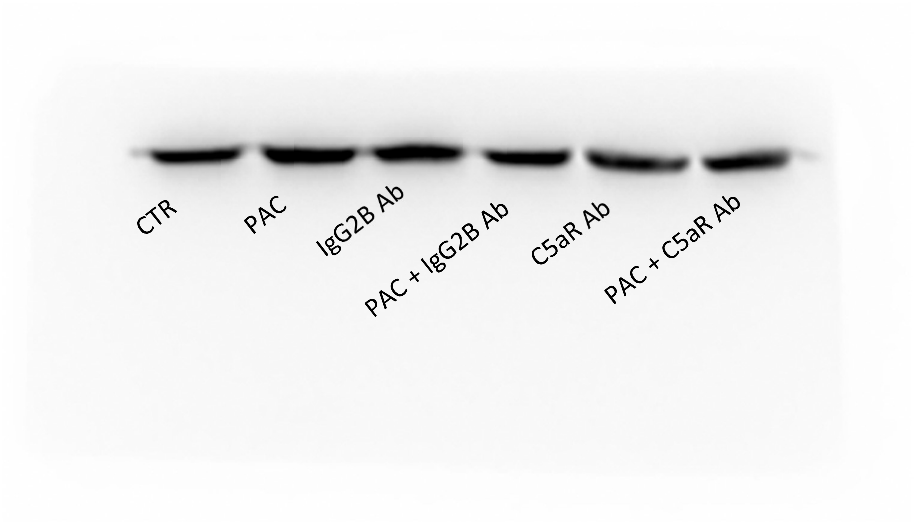

P-Jak2

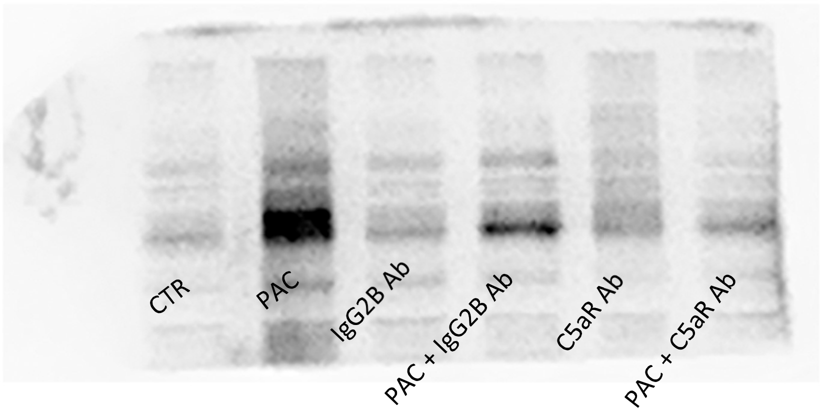

Actin

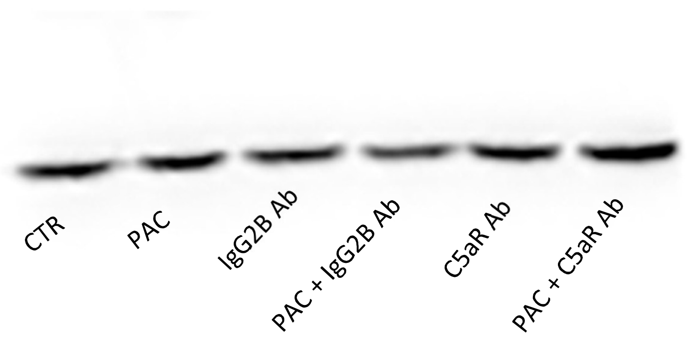

Supplement: Supplementary file 2 — Supplemental material [file 41419_2022_4964_MOESM2_ESM.pdf]
