# Supplementary material for: Paclitaxel binds and activates C5aR1: A new potential therapeutic target for the prevention of chemotherapy-induced peripheral neuropathy and hypersensitivity reactions
Source: Cell Death Dis. 2022 May 25;13(5):500. doi: 10.1038/s41419-022-04964-w (PMC9130998; doi:10.1038/s41419-022-04964-w)
Supplement: Supplementary file 4 — Revised Manuscript - marked up [file 41419_2022_4964_MOESM4_ESM.docx]

*Article*

**Paclitaxel binds and activates C5aR1: a new potential therapeutic target for the prevention of chemotherapy-induced peripheral neuropathy and hypersensitivity reactions**

Brandolini Laura^1^*^*^*, d’Angelo Michele^2^*^*^*, Novelli Rubina^3^, Castelli Vanessa^2^, Giorgio Cristina^4^, Sirico Anna^4^, Cocchiaro Pasquale^4^, D’Egidio Francesco^2^, Benedetti Elisabetta^2^, Cristiano Claudia^5^, Bugatti Antonella ^6^, Ruocco Anna ^4^, Amendola Pier Giorgio^4^, Talarico Carmine^4^, Manelfi Candida ^4^, Iaconis Daniela ^4^, Beccari Andrea^4^, Quadros Andreza U^7^, Cunha Thiago M^7^, Caruso Arnaldo ^6^, Russo Roberto^5^, Cimini Annamaria^2,8^, Aramini Andrea^1^, Allegretti Marcello^1^

^1^Dompé Farmaceutici SpA, Via Campo di Pile, 67100 L’Aquila, Italy

^2^Department of Life, Health and Environmental Sciences, University of L’Aquila, 67100 L’Aquila, Italy

^3^ Dompé Farmaceutici SpA, Via S. Lucia, 20122 Milan, Italy

^4^ Dompé Farmaceutici SpA, Via De Amicis, 80131 Naples, Italy

^5^ Department of Pharmacy, University of Naples Federico II, 80131 Naples, Italy

^6^ Department of Molecular and Traslational Medicine, University of Brescia Medical School, 25123 Brescia, Italy

^7^ Center for Research in Inflammatory Diseases (CRID), Department of Pharmacology, Ribeirao Preto Medical School, University of Sao Paulo, Ribeirão Preto - SP, 14049-900, Brazil

^8^Sbarro Institute for Cancer Research and Molecular Medicine and Center for Biotechnology, Temple University, Philadelphia, PA 19122, USA

*^*^* These authors contributed equally to this work

**Running title: C5aR1 mediates paclitaxel neuropathy and anaphylaxis**

*Corresponding author*

Marcello Allegretti

marcello.allegretti@dompe.com

**ABSTRACT**

Chemotherapy-induced peripheral neuropathy (CIPN) and hypersensitivity reactions (HSRs) are among the most frequent and impairing side effects of the antineoplastic agent paclitaxel. Here, we demonstrated that paclitaxel can bind and activate complement component 5a receptor 1 (C5aR1) and that this binding is crucial in the etiology of paclitaxel-induced CIPN and anaphylaxis. Starting from our previous data demonstrating the role of interleukin (IL)-8 in paclitaxel-induced neuronal toxicity, we searched for proteins that activate IL-8 expression and, by using the Exscalate platform for molecular docking simulations, we predicted the high affinity of C5aR1 with paclitaxel. By *in vitro* studies, we confirmed the specific and competitive nature of the C5aR1-paclitaxel binding and found that it triggers intracellularly the NFkB/P38 pathway and c-Fos. In F11 neuronal cells and rat dorsal root ganglia, C5aR1 inhibition protected from paclitaxel-induced neuropathological effects, while in paclitaxel-treated mice, the absence (knock-out mice) or the inhibition of C5aR1 significantly ameliorated CIPN symptoms - in terms of cold and mechanical allodynia - and reduced the chronic pathological state in the paw. Finally, we found that C5aR1 inhibition can counteract paclitaxel-induced anaphylactic cytokine release in macrophages *in vitro*, as well as the onset of HSRs in mice.

Altogether these data identified C5aR1 as a key mediator and a new potential pharmacological target for the prevention and treatment of CIPN and HSRs induced by paclitaxel.

1. **INTRODUCTION**

Extracted by the bark of the Pacific Yew Tree, *Taxus brevifolia*,(1) paclitaxel (Taxol®) is a member of the taxane family of anticancer drugs commonly used for the treatment of ovarian, breast, and non-small cell lung cancer, as well as AIDS-related Kaposi's sarcoma(2).

Although highly efficient in inhibiting tumor growth, paclitaxel is generally not well tolerated, and its use is sometimes limited because of the occurrence of serious adverse drug reactions(3). Among these, hypersensitivity reactions (HSRs) and peripheral sensory neuropathy are some of the most impairing ones. HSRs, ranging from mild to lethal, occur within the first few minutes of administration, especially during the first or second exposure, and can affect up to 40% of patients(4). Premedication with corticosteroids, antihistamines and H2 antagonists can reduce the incidence of mild to moderate HSRs, and desensitization protocols have shown to be generally safe and efficient approaches; however, premedication does not protect against severe HSRs (i.e. anaphylaxis)(4) and some patients still experience breakthrough reactions during desensitization protocols(5). Chemotherapy-induced peripheral neuropathy (CIPN), on the other hand, is a debilitating, mostly sensory neuropathy, result of progressive and often irreversible damage of the peripheral nervous system. Its prevalence reaches 60-70% in patients receiving paclitaxel(6), and despite scientists’ efforts during the last decades, no single effective therapeutic approach is currently available for preventing or treating CIPN(7).

Several studies have connected HSRs with the induction of inflammation by paclitaxel(8), which is known to trigger the production of proinflammatory cytokines such as interleukin (IL)-8(9) and the activation of the complement cascade. Besides HSRs, inflammation has been associated also with paclitaxel-induced CIPN(7), and IL-8 in particular emerged as a crucial factor in paclitaxel-induced neuronal toxicity, which can be reduced by the inhibition of IL-8 receptors CXCR1 and CXCR2(10,11). Thus, the IL-8 pathway has been proposed as a potential pharmacological target for the treatment of CIPN(10), and this prompt us to investigate IL-8 upstream events that could be involved in paclitaxel-induced CIPN and anaphylaxis to eventually develop effective strategies that are urgently needed for the prevention and treatment of these side effects of paclitaxel.

C5a, the anaphylatoxin produced following complement activation, is known to mediate potent nociceptive effects mainly by interacting with the G-protein coupled receptor (GPCR) complement component 5a receptor 1 (C5aR1)(12), and the activation of the C5a-C5aR1 axis has been associated with neuropathic pain(13) and neuro-inflammatory injury(14). Here, we demonstrate that paclitaxel binds and activates C5aR1, and this binding is crucial in the etiology of paclitaxel-induced CIPN and anaphylaxis. Thus, we identify C5aR1 as a new potential target for the prevention and treatment of anaphylaxis and CIPN in patients receiving paclitaxel.

1. **RESULTS**
   1. ***Screening of the identified receptors and prediction of C5aR1-paclitaxel binding***

To identify *in silico* the best paclitaxel potential targets among those selected by bioinformatic analysis and literature search (for details, Supplementary information paragraph 1), our in-house Comprehensive Therapeutic Target Database (CTTD), the Protein Data Bank (PDB) database, and AlphaFold models were used to retrieve 3D protein structures of the receptors previously selected (Table 1).

All the 3D protein structures were prepared using the Protein Preparation Wizard of Schrodinger, while docking of taxol was performed using LiGen™(15) in each binding site. In the case of GPCRs, when the structures were only available in apo form (with no ligand), the binding sites were identified by alignment on other GPCRs in holo form. From the Exscalate-driven molecular docking simulations, we obtained that 6 of the analyzed proteins were predicted to have high affinity with paclitaxel, with an imbalance in favor of the GPCR class (Table 1).

Among the proteins with predicted high affinity, C5aR1 was one of the best scored candidates. Taking advantage of the analysis of X-ray structures and the information that we gained from our previous studies(16), we investigated the potential binding between C5aR1 and paclitaxel and identified three main binding sites of C5aR1, one orthosteric and two allosteric. To get a focus on some key points of orthosteric and allosteric binding sites, we chose the 6C1R pdb model for the molecular docking experiments, and because of the higher resolution of the model, this crystal structure was selected for further computational studies and submitted to a structural and chemical refinement to increase the reliability of the protein. Performing the docking of paclitaxel on 6C1R crystal structure in the orthosteric binding site, we found an interesting binding pose and several interactions with Arg175, Val190, Tyr192 Tyr258, Phe275, Lys279, and Asp282 key residues of the binding site (Figure 1A and B). This was unexpected as C5aR1 was not present among the already known targets of paclitaxel, as confirmed also by the target list retrieved from GoStar database (GVK BIO Online Structure Activity Relationship Database) (Supplementary Information).

Based on our analysis and considering the role of C5a-C5aR1 axis in immune reactions and CIPN, we selected C5aR1 for further investigations of its binding with paclitaxel and its potential involvement in paclitaxel-induced CIPN.

- 1. ***In vitro studies confirmed paclitaxel-C5aR1 binding***

To validate our *in silico* prediction, we performed Surface Plasmon Resonance (SPR) analysis(17). Recombinant human C5aR1 was immobilized over a CM5 sensorchip, while paclitaxel was let to flow across it. Results revealed that paclitaxel can indeed bind to immobilized C5aR1 (Figure 2A). The binding is dose-dependent, displaying a Kd value of 670 nM (Figure 2B) and an affinity value at equilibrium obtained from measurement of steady-state binding levels of 525 nM (Figure S1A). Data obtained *in silico* by comparing paclitaxel binding mode to C5aR1 and C5aR2, revealed that paclitaxel cannot bind C5aR2 binding site through the same extended interaction pattern observed for C5aR1 (Figure S1B). Confirming C5aR1-paclitaxel binding selectivity, we found that paclitaxel was not able to bind to immobilized C5aR2, C5a and C5a des-Arg proteins (Figure 2A).

To deepen the investigation and further understand the interaction between paclitaxel and C5aR1, we then co-injected C5aR1 (50 nM) with increasing concentrations of paclitaxel on C5a or C5a des-Arg surface. In this setting, paclitaxel was able to inhibit the binding of C5aR1 to C5a or to C5a des-Arg in a dose-dependent manner with a half maximal inhibitory concentration (IC_50_) value of 4.579 µM (Figures 2C and D) and of 2.134 μM (Figures 2E and F), respectively. To confirm the capacity of paclitaxel to bind specifically to the binding site of C5aR1 for its natural ligand C5a, we performed competition experiments using a monoclonal anti-C5aR1 antibody, which is known to neutralize the C5aR1 and C5a interaction (Figure S1C), and observed that paclitaxel could inhibit the C5aR1/anti-C5aR1 interaction in a dose dependent manner with an IC_50_ value of 3.632 µM (Figures 2G and H). To define whether paclitaxel induces the activation or the inhibition of C5aR1 through its binding, we then performed a cell-based cAMP assay in single dose experiments and found that paclitaxel can activate, but not inhibit, C5aR1, while dose-response experiments showed that paclitaxel EC_50_ is 5.81 µM and Emax around 50%, demonstrating that paclitaxel is a new partial agonist of C5aR1 (Figure S1D).

Taken together, these results demonstrate that paclitaxel can competitively and specifically bind and activate C5aR1, thus suggesting the potential involvement of C5aR1 in the mediation of paclitaxel side effects. Notably, we also performed SPR experiments to investigate whether also docetaxel, another chemotherapeutic agent of the taxane family(18), could interfere with the interaction between C5aR1 and C5aR2 and their natural ligands (C5a and C5a des-Arg). With these experiments (for details, Supplementary information paragraph 2), we demonstrated that, similarly to paclitaxel, also docetaxel can bind C5aR1 receptor in a dose dependent way (Figure S2A-D).

- 1. ***C5aR1 is a key mediator of paclitaxel-induced CIPN in vitro and vivo***

Having identified C5aR1 as a new target of paclitaxel, we investigated the intracellular pathways that could be activated following the binding and the potential involvement of C5aR1 in paclitaxel-induced neurotoxic effects. *In vitro* experiments in F11 cells showed that C5aR1 binding by paclitaxel induces the activation of NFkB/p38 pathway and c-Fos (for details, Supplementary information paragraph 3, and Figures 3 and S3A) and that C5aR1 inhibition significantly protected from paclitaxel neuropathological effects *in vitro* (for details, Supplementary information paragraph 4, and Figures 4, S3B, S4 and S5). Moreover, C5aR1 inhibition completely restored the altered electrical activity of paclitaxel-treated dorsal root ganglia (DRG) (for details, Supplementary information paragraph 5, and Figure 5), further demonstrating the crucial role of C5aR1 in the neuropathological mechanisms underlying paclitaxel-induced CIPN.

Then, to investigate the role of C5aR1 in paclitaxel-induced CIPN *in vivo*, wild-type and *C5ar1* knock-out (KO) mice were treated intraperitoneally with paclitaxel at 4 mg/kg for four times on alternate days (days 1, 3, 5, and 7)(19), and CIPN symptoms in terms of cold and mechanical allodynia were evaluated after the injection of paclitaxel. As expected, paclitaxel administration induced cold allodynia in wild-type animals from day 3 until day 14 compared to sham mice (Figure 6A). Paclitaxel induced also evident allodynia to mechanical stimuli at all time points (day 1, 3, 5, 7, 10, and 14) compared to sham mice (Figure 6B). Notably, the absence of C5aR1 in KO mice was significantly protective during both the induction and maintenance phases of paclitaxel-induced CIPN (Figure S6), thus indicating the crucial role of C5aR1 in the pathophysiology of paclitaxel-induced CIPN.

Starting from these data, we evaluated the potential therapeutic effects of C5aR1 pharmacological inhibition. Pharmacological inhibition of C5aR1 with oral DF3966A at 30 mg/kg administered every 12 hours for 14 days during the induction phase of CIPN significantly reduced cold allodynia induced by paclitaxel at day 3 and 5, and at day 7, 10 and 14 (Figure 6A), and significantly inhibited paclitaxel mechanical allodynic effect from day 3 until day 14 (Figure 6B). These data clearly demonstrate the key role of C5aR1 in mediating paclitaxel-induced CIPN and the potential therapeutic effects of the inhibition of C5aR1 in this condition.

To explain the mechanisms underlying the protective effects of the inhibition of C5aR1 in paclitaxel-induced CIPN, we analyzed by real time-PCR the gene expression of C5aR1, inflammatory markers, and TRPV1 and TRPV4 in mouse spinal cord and paw samples collected at day 15 after the first paclitaxel administration. In the spinal cords of paclitaxel-treated mice, C5aR1 gene expression was significantly increased as compared with sham mice, and this upregulation was completely inhibited by treatment with DF3966A (Figure 6C). Similarly, DF3966A significantly reduced the paclitaxel-induced increase of mRNA level of TNF-α, a mediator of both spinal microglial activation and hypersensitivity to neuropathic pain(20) (Figure 6C). In paws, C5aR1 mRNA level was not affected significantly by paclitaxel alone or paclitaxel+DF3966A treatment, but TRPV1, TRPV4, IL-1β and IL-6 mRNA levels were upregulated in mice treated with paclitaxel, and DF3966A treated-mice showed a significant reduction of their levels (Figure 6D). These results suggest that the central (spinal cord) activation of C5aR1 mediated by paclitaxel is crucial for the modulation of peripheral mediators (inflammatory cytokines), which in turn amplify pain transmission.

- 1. ***C5aR1 inhibition counteracts paclitaxel- or C5a-induced anaphylactoid cytokine release in macrophages***

Considering the role of C5a-C5aR1 axis in anaphylactoid reactions of different origin (21,22), we hypothesized that C5aR1 could be involved also in paclitaxel-induced anaphylaxis. Since macrophages play a crucial role in both CIPN(23) and anaphylaxis(24), express C5aR1 and have been used to study the role of C5a/C5aR pathway in different conditions(25,26), we used mouse (RAW 264.7) and human (THP-1) macrophages to investigate whether C5aR1 could mediate paclitaxel-induced anaphylactoid reactions. We performed the profiling of cytokines released (> 40 cytokines) from M0 (differentiated, not stimulated) RAW 264.7 and human macrophages challenged with paclitaxel (10 nM) or C5a (10 nM) for 24h using a proteome profiler assay. Upon both treatments, murine and human proteome profiles resembled that of typical anaphylaxis and overlapped for all cytokines analyzed, excluding C5a that was released only by C5a-stimulated cells, as expected (Figure 7). In paclitaxel- and C5a-treated RAW 264.7, the levels of monocyte chemoattractant protein-1 (MCP-1), which is a chemokine involved in basophil migration and anaphylaxis(27), resulted significantly increased compared to the control group, as well as those of macrophage inflammatory protein (MIP)-1α and MIP-1β, and of MIP-2 and IL1Ra (Figure 7A-B). The release of these cytokines was counteracted by C5aR1 inhibition mediated by Avacopan (1μM, used as C5aR1 inhibitor reference standard(28)), or by an anti-C5aR1 antibody (1μM), or by DF3966A (1μM) (Figures 7A-B).

The analysis on human macrophages confirmed the data obtained with murine ones as the profile of cytokine released upon C5a overlapped with that obtained upon paclitaxel stimulus, and also in these experimental conditions, DF3966A, Avacopan and C5aR1 antagonist effectively counteracted the C5a- and paclitaxel-induced cytokine release (Figures 7C-D). Notably, both paclitaxel and C5a stimulated also the release of IL-8 by human macrophages, and this effect was again counteracted by Avacopan, the anti-C5aR1 antibody, and DF3966A (Figures 7C-D).

Altogether these data indicate the potential involvement of C5aR1 in mediating also paclitaxel-induced anaphylactoid reactions and led us to evaluate C5aR1 role in these conditions *in vivo*.

***2.5 C5aR1 inhibition prevents paclitaxel-induced anaphylactoid reactions in vivo***

Having identified paclitaxel as the key factor responsible for anaphylactoid reactions induced by the chemotherapy treatment (for details, Supplementary information paragraph 6 and Figure S7), we then investigated the potential effects of the inhibition of C5aR1 as preventive treatment for paclitaxel-induced HSRs. C5aR1 inhibition was achieved treating animals with DF3966A (30 mg/kg, os) or with Avacopan (30 mg/kg, os) administered respectively 3 hours and 1 hour before paclitaxel administration, according to their pharmacokinetic profile (Supplementary information paragraphs 7 and 8 and (29)). Pharmacological inhibition of C5aR1 by both treatments effectively counteracted the onset of HSRs abolishing paclitaxel-induced increase of vascular leakage and plasma extravasation (Figures 8A and B). Moreover, pre-treatment with DF3966A or Avacopan significatively reduced the increase of serum concentration of both histamine and complement 5b-9 (SC5b-9) (Figures 8C and D), which is associated with increased activation of the complement pathway, in paclitaxel-treated mice, suggesting a key role of C5aR1 in the early first phase of inflammation in HSRs induced by paclitaxel and supporting its inhibition as a potential pharmacological strategy for the prevention of paclitaxel-induced anaphylaxis.

**DISCUSSION**

For cancer patients undergoing paclitaxel treatment, CIPN and HSRs are frequent adverse drug reactions that severely impair patient’s quality of life(4). In this study, we reveal the previously unknown role of C5aR1 as target of paclitaxel and provide the first demonstration that C5aR1 is a mediator of both CIPN and anaphylactoid reactions induced by paclitaxel and that the inhibition of this receptor can counteract these side effects *in vitro* and *in vivo*.

Numerous studies have indicated the pro-inflammatory effects of paclitaxel, and in particular its stimulation of IL-8 expression, as crucially involved in the onset and maintenance of paclitaxel-induced neuropathy(10,11). Starting from these data and with the aim of finding new possible targets for the prevention and treatment of CIPN induced by paclitaxel, we took advantage of our supercomputing platform (EXaSCale smArt pLatform Against paThogEns (EnsEXSCALATE))(30), to identify IL-8 upstream mediators that could possibly bind paclitaxel and mediate its side effects. Surprisingly, C5aR1 emerged as one of the receptors displaying the best predicted affinity for paclitaxel, and its high affinity was confirmed by *in vitro* SPR studies and competition experiments. By applying the same technology used to screen and test several molecules with potential efficacy against SARS-CoV-2(30) and combining it to the concept that drives drug repurposing, we thus successfully “deorphanized” C5aR1 for paclitaxel and identified a new target for this drug that can mediate some of its side effects.

The binding of paclitaxel to C5aR1 results in the direct activation of the NFkB/P38 pathway and of c-fos/c-Jun, which are known to be upstream of the IL-8 pathway and involved in its activation(31), thus suggesting that C5aR1 could be involved in paclitaxel-induced CXCL1-mediated neurotoxic effects on F11 sensory neurons that we observed at 24 hours in previous studies (10). The corroboration of the role of the C5aR1-CXCL1 pathway in F11 toxicity was given by the altered expression of p-FAK, p-cortactin and the JAK-STAT pathway, as well as of CXCL1, observed in F11 cells after the administration of paclitaxel and C5a. Notably, phosphorylation time-point experiments indicated that these effects are primary C5aR1-mediated responses, on which CXCL1/IL-8 acts later in time to further sustain these changes and make them chronic. These alterations were successfully corrected by the treatment with an antibody against C5aR1 (C5aR1 Ab) or with a C5aR1 antagonist, thus demonstrating the crucial role of C5aR1 in the mediation of paclitaxel neurotoxicity *in vitro* and that the inhibition of C5aR1 can effectively counteract paclitaxel downstream neuropathological effects.

The complement system is known to play a crucial role in chronic pain(32). However, whether and how the complement, and C5a-C5aR1 axis in particular, participates in CIPN is still unclear in the literature. *In vivo*, the significant reduction of paclitaxel-induced allodynia that we observed in C5aR1 knock-out mice and after treatment with a C5aR1 inhibitor upon paclitaxel administration demonstrated that C5aR1 is crucially involved in the mediation of paclitaxel neuropathy and suggested the potential therapeutic effects of the inhibition of C5aR1 to treat or prevent this condition. Further confirming these results, we observed that paclitaxel and C5a exhibited comparable effects on the electrophysiological behaviors of DRG primary neurons, and C5aR1 inhibition completely counteracted both paclitaxel- and C5a- induced alterations.

Recently, an interesting study, which described the reduction of paclitaxel-induced mechanical allodynia in complement component 3 (C3) knockout rats compared to normal animals, thus suggesting that complement might be a novel target for the treatment of CIPN(33). Building on these data, in 2020 the National Institutes of Health (NIH) has funded a new study to explore the role of the complement system in CIPN with the goal of identifying new pathways for treatment. With our data, we have now defined the role of complement system in paclitaxel-induced CIPN demonstrating that *i)* paclitaxel can directly bind and activate C5aR1, *ii)* the activation of C5aR1 is crucial for the mediation of paclitaxel-induced CIPN, and *iii)* the inhibition of C5aR1 effectively counteracts CIPN. As limited options are available to effectively treat CIPN once it is established(34), a pharmacological approach, such as the inhibition of C5aR1, able to prevent CIPN from developing, can open an entirely new scenario for the prevention of this frequent and severely impairing side effect.

Given the role of C5a, the strongest anaphylatoxin(35), and of C5aR1 as critical regulators of asthma(36) and oral antigen-induced anaphylaxis(21), we then hypothesized the involvement of C5aR1 also in another paclitaxel-induced side effect, HSRs and anaphylaxis. Corroborating our hypothesis, murine RAW264.7 and human THP-1 macrophages displayed a superimposable anaphylactoid proteome profile when treated with paclitaxel or with C5a, and the cytokine release induced by both treatments was counteracted by C5aR1 inhibition. Notably, the use of human THP-1 cells allowed us to evaluate also the contribution of NLRP3 inflammasome in treatment-induced responses, and this is of crucial importance given the inflammasome key role in immune reactions, its interaction with the complement pathway and the fact that it can be activated by paclitaxel (8,37,38).

Having assessed that paclitaxel *per se*, and not its vehicle Cremophor EL, is the main responsible for anaphylactoid reactions induced by the chemotherapy treatment *in vivo*, we demonstrated that C5aR1 as a crucial factor in the pathophysiology of HSRs induced by paclitaxel and can thus be a potential pharmacological target for their prevention and blockade. These results, together with those obtained in CIPN, are even more significant considering that also treatment with other taxanes (i.e., docetaxel) and next-generation taxanes (i.e. nab-paclitaxel, an albumin-bound solvent-free paclitaxel formulation) is associated with the development of CIPN and HSRs(39–41).

Since the importance of complement system in cancer biology has been demonstrated, the role of C5a-C5aR1 axis in the pathogenesis of different types of cancer has been explored in several studies along with the potential therapeutic effect of its inhibition. In particular, the effects of C5a deficiency and C5aR1 inhibition have been evaluated in mice with colorectal(42) and lung cancer(43), demonstrating the efficient reduction of tumor size and the enhancement of the effects of anti-cancer chemotherapy. Moreover, preclinical studies have also suggested an additive effect on tumor size reduction of anti-complement drugs with immune checkpoint inhibitors, possibly due to the inhibitory effect of complement blockade on myeloid cell recruitment and polarization(43,44). In combination with paclitaxel, several immunotherapies have been already tested in preclinical and clinical trials for the treatment of different types of tumor and showed promising results improving paclitaxel antitumoral activity(45–47). Thus, although the combination of anti-complement therapies with other anti-cancer agents should be further investigated, the increasing number of studies that demonstrate the improved antitumor responses obtained combining complement inhibition – and in particular C5aR1 inhibition – with chemotherapy and/or immunotherapy, together with our present results on paclitaxel-mediated C5aR1 activation, further sustain that a combined treatment with paclitaxel and C5aR1 inhibitors could be a promising therapeutic approach, not only to prevent CIPN and HSRs, but also to potentially obtain a synergistic effect on tumor progression.

In conclusion, in this study, we identified C5aR1 as a new target of paclitaxel and as the mediator of its related CIPN and HSRs, demonstrating that C5aR1 inhibition can counteract both these side effects. These findings are groundbreaking, as not only they shed light on the mechanisms that underlie the pathophysiology of CIPN and HSRs due to paclitaxel treatment, but also indicate a potential pharmacological target for their prevention, opening new possibilities for the management of the continuously growing number of patients that still experience serious long-term consequences or even death(4,48) following the treatment with paclitaxel.

**MATERIALS AND METHODS**

***Bioinformatic analysis***

Enrichment analysis was performed using MetaCore^TM^ version 21.3 build 70600, a Cortellis solution from Clarivate. MetaCore is an integrated knowledgebase and software suite for pathway and network analysis of high throughput transcriptomic data. It contains 1,456 protein interaction pathways, which are a comprehensive resource of human, mouse, and rat signaling, metabolism, diseases, and stem cells, all manually curated from peer-reviewed literature. We queried the database with the IL-8 human protein and look for Interaction with the following parameters: Direction – Incoming; Effect – Activation; Mechanism – Influence on expression and Transcriptional regulation. Also, GOSTAR (GVK Biosciences Private Limited, Plot No. 28 A, IDA Nacharam, Hyderabad, India, 2010. https://gostardb.com/gostar/), an online scientific database product of Excelra Knowledge Solutions consisting of published and patented inhibitors against various biological targets and their associated structure-activity relationship (SAR) data, was used to retrieve known targets of paclitaxel.

***Functional assay***

A cell-based cAMP assay was provided by DiscoverX (cod. 86-0007P-2277AG for C5aR1 and 86-0007P-2818AG for C5aR2). The assay utilizes a cell line stably expressing non-tagged GPCRs. The activation of a GPCR via Gi and Gs secondary messenger signaling was measured by Hit Hunter® cAMP assay in a homogenous, non-imaging assay format using a technology developed by DiscoverX called Enzyme Fragment Complementation (EFC) with β-galactosidase (β-Gal) as the functional reporter. The IC50 was calculated from concentration-effect-curves after non-linear regression analysis.

***SPR binding assay***

SPR measurements were performed on a BIAcore X instrument (Cytiva). For the study of paclitaxel or docetaxel and C5aR1 or C5aR2 interaction, anti-c-Myc antibodies or anti-Histidine antibodies were immobilized onto a CM5 sensorchip using standard amine-coupling chemistry. Then, recombinant human C5aR1 with a C-terminal c-Myc tag or recombinant human C5aR2 with a C-terminal 6xHistidine tag (10 µg/ml in 50mM HEPES, pH 7.0, containing 0.01% cholesteryl hemisuccinate Tris salt, 0.1% CHAPS, and 0.33 mM synthetic phospholipid blend (dioleoyl) DOPC:DOPS (7:3 (w/w) (running buffer), Avanti Polar Lipids, Alabaster, AL, USA) were injected over the specific antibodies surfaces, allowing the immobilization of 849 RU or 1190 RU of receptors, respectively. A sensor chip coated with anti-c-Myc antibodies or anti-Histidine antibodies alone was used as a negative control and for blank subtraction. For the study of paclitaxel or docetaxel and C5a or C5a des-Arg interaction, 20 ug/ml of human recombinant proteins were immobilized onto a CM5 sensorchip using standard amine-coupling chemistry, allowing the immobilization of 1913 RU or 1520 RU, respectively. Lastly, for the study of paclitaxel or docetaxel and anti-C5aR1 antibody (CD88 (S5/1)) interaction, 20 ug/ml of monoclonal antibody was immobilized onto a CM5 sensorchip using standard amine-coupling chemistry, allowing the immobilization of 4015 RU. The screening of the capacity of paclitaxel or docetaxel to bind to C5aR1/2 receptors or to C5a/C5a des-Arg proteins was performed in single cycle analysis as follows: paclitaxel or docetaxel were resuspended at a final concentration of 200 μM in running buffer (for analysis on C5aR1 and C5aR2) or in 10 mM HEPES, pH 7.4 containing 150 mM NaCl, 3 mM EDTA, and 0.005% surfactant P20 (HBS-EP+) (for analysis on C5a and C5a des-Arg), and were injected over the different surfaces for 2 min and then washed until dissociation.

For determination of the kinetic parameters of paclitaxel or docetaxel on C5aR1 surface, increasing concentrations of the compound in running buffer were injected over the C5aR1 or control surfaces for 2 min and then washed until dissociation. Kinetic parameters were calculated from the sensorgram overlays by using the nonlinear fitting single site model software package BIAevaluation (version 3.2). Only sensorgrams whose fitting gave χ2 values close to 10 were used (1). Also, Kd was calculated by being fitted with the proper form of Scatchard’s equation for the plot of the bound RU at equilibrium versus the ligand concentration in solution. For competition experiments, C5aR1 (50 nM) and increasing concentrations of paclitaxel or docetaxel were injected over the C5a, C5a des-Arg and CD88 surfaces. The reagents that have been used are: recombinant human C5a anaphylotoxin chemotactic receptor 2 (C5AR2) (CUSABIO TECHNOLOGY, Houston, TX, US), recombinant protein of human complement component 5a receptor 1 (C5AR1) (Origene, Rockville, MD, US), anti-6X His tag antibody (Abcam, Cambridge, UK), Anti-Myc/c-Myc Antibody (9E10) (Santa Cruz Biotechnology, Dallas, Texas, US) and CD88 (clone S5/1) antibody (sc-53795, Santa Cruz Biotechnology), complement C5a des-Arg, Human (Merck, [Burlington, Massachusetts,](https://www.google.com/search?client=firefox-b-d&q=Burlington+(Massachusetts)&stick=H4sIAAAAAAAAAOPgE-LSz9U3MKoyLjMtUuIEsQ1zjXILtbSyk63084vSE_MyqxJLMvPzUDhWGamJKYWliUUlqUXFi1ilnEqLcjLz0kvy8xQ0fBOLixOTM0qLU0tKijV3sDLuYmfiYAAAoTofEGoAAAA&sa=X&ved=2ahUKEwiupvSE_IP3AhU7RPEDHfjBBCIQmxMoAXoECEEQAw) USA).

***Cell cultures***

All cell cultures were routinely tested for Mycoplasma contamination using Mycoplasma PCR Detection Kit, Applied Biological Materials, USA. The F11 hybridoma cells (ECACC 08062601), chosen as an alternative model of DRG neurons(10) were cultured in DMEM (Euroclone, MI, Italy) medium supplemented with 10% FBS (Sigma-Aldrich St. Louis, CO, USA), 1% penicillin/streptomycin (Euroclone) and 1% glutamine (Euroclone) at 37°C, in a humidified 95% air-5% CO_2_ atmosphere. For all the experiments, cells were used at 18^th^ passage and seeded at 1 x 10^4^ cells/cm^2^. After 24h, cells were differentiated with rat NGF (rNGF) (Sigma). rNGF was dissolved in DMEM with 1% penicillin/streptomycin and 1% glutamine (FBS free) at the final concentration 50 ng/ml. The medium was replaced every 3 days until complete differentiation, which happened after 7 days.

DRG neurons were obtained by dissociation of postnatal (P2) Sprague Dawley rat ganglia (Envigo, Bresso Italy), following well-defined protocols(49,50). To minimize introduction of contaminating cells, any excessively long roots were trimmed from DRGs. After the isolation, DRGs were enzymatically dissociated as follows: 20 minutes of 1.5 mg/ml papain exposure at 37°C and 30 minutes of 4mg/ml collagenase exposure at 37°C. Subsequently, cells were mechanically dissociated with a fire-polished glass Pasteur pipette to obtain a single-cell suspension. Once dissociation was achieved, cells were seeded onto 96 black multiwell plates coated with Laminin (10ug/mL, overnight at Room Temperature, RT) at selected densities. Following 1 week of incubation in DMEM high glucose medium supplemented with 10% FBS, 1x N2, 1x B27, 100 IU/ml penicillin, 10 mg/ml streptomycin, cells were treated according to experimental needs.

RAW 264.7 (ATCC TIB-71) mouse macrophages were cultured following manufacturer’s instructions using DMEM supplemented with 10% FBS (non-heat inactivated) (ATCC, USA) and then were seeded at 1x10^4^ cells/cm2. After 24 hours, cells were treated with C5a (diluted in medium) (10 nM, R&D, USA) or PAC (diluted in medium) (10 nM, Sigma, USA) for 24 hours, then the media was replaced with culture media (5ml) for 24h and finally collected for proteome profiler array.

Human monocytes THP-1 (ATCC TIB-202) were purchased from ATCC and cultured following manufacturer’s instructions in RPMI supplemented with FBS 10% (non-heat activated), 1% Glut and 2-mercaptoethanol to a final concentration of 0.05 mM. Then, THP-1 were differentiated using 20 ng/ml phorbol 12-myristate 13-acetate (PMA) for 72 hours. Cells were then treated with the different treatments as described below. Media was replaced with culture media (5ml) for 24h and then collected for proteome profiler array.

***Drug treatments***

Paclitaxel stock solution (Sigma-Aldrich; 10mM) was prepared by dissolving the powder in DMSO and aliquots were stored at -20°C. C5a stock solution (R&D Systems, Inc. MN, USA; 20 mM) was prepared by dissolving the powder in DMSO 100%; aliquots were stored at -80°C. IgG2B Ab, C5aR Ab formulation (BioLegend, CA, USA) or C5aR1 antagonist (MBS691011, MyBioSource, USA) Avacopan (Selleckchem, USA) were supplied ready to use. DF3966A (Dompé farmaceutici S.p.A., L'Aquila, Italy) at stock solution 20 mM was prepared in DMSO 100%.

***Western blotting***

F11 cells were treated for 24 hours with paclitaxel (10nM final concentration), C5a (10nM final concentrations) or IgG2B Ab or C5aR Ab (50nM final concentration), or the combination of each antibody with paclitaxel or DF3966A (10µM final concentration). For the time points differentiated F11 were treated for shorter times, such as 15 minutes, 30 minutes and 90 minutes with paclitaxel (10nM) or C5a (10nM). Control and treated cells were collected and lysed in ice-cold RIPA buffer (phosphate buffer saline pH 7.4 containing 0.5% sodium deoxycholate, 1% Igepal P-40, 0.1% SDS, 5mM EDTA, 1% protease and phosphatase inhibitor cocktails, Sigma-Aldrich, USA). Protein lysates (30µg) were separated on Bolt precast 4-12% SDS–polyacrylamide gel and electroblotted onto polyvinyldifluoride membrane (PVDF; Thermo, USA). Nonspecific binding sites were blocked using 5% Blotto dry milk (Santa Cruz, USA) diluted in 1x Tris-buffered saline with Tween 20 (ChemCruz, USA) for 1 hour at RT. To evaluate C5aR1 expression, control membranes were then incubated overnight at 4°C with rat C5aR1 1:200 (sc-53788, Santa Cruz Biotechnology, Inc. Dallas, Texas, USA), diluted with TBS containing 0,1% Tween 20 (TBS-T) and 5% non-fat dry milk. As secondary antibodies, peroxidase conjugated anti-Rat IgG (1:10000; Sigma) was used. For the other detected proteins, membranes were incubated overnight at 4°C with the following primary antibodies, diluted in the blocking solution: rabbit anti p-Fak 1:1000 (Tyr397, 3283S, Cell Signalling, USA), rabbit anti p-STAT3 1:1000 (Tyr705, 9131S, Cell Signaling, USA), rabbit anti p-cortactin 1:500 (Abcam, UK), rabbit anti cortactin 1:500 (ab81208), rabbit anti-JAK2 (3230S, Cell Signaling), anti-p-Jak2 1:200 (Tyr 1007/Tyr 1008, sc16566, Santa Cruz, USA), rabbit anti p-NFKB 1:1000 (Ser536, ab239882, Abcam), rabbit anti NFKB 1:1000 (ab16502, Abcam), rabbit anti p-c-fos (5348T, Ser325348, Cell Signaling), rabbit c-fos 1:1000 (2250T, Cell Signaling), rabbit anti-p-jun 1:1000 (T93, ab81319, Abcam), anti-c-jun 1:1000 (ab32137, Abcam), rabbit anti p-P38 1:1000 (Thr180/Tyr182, 9211S, Cell Signaling), rabbit anti-p38 1:1000 (9212, Cell Signaling), rabbit anti-p-MAPK6/ERK3 1:1000 (S189, ab74032, Abcam), rabbit anti-MAPK6/ERK3 1:1000 (ab53277, Abcam). As secondary antibodies, peroxidase-conjugated anti-rabbit or anti-mouse IgG (1:30000; Invitrogen, USA) were used. To reprobe the same membrane, Restore Western Blot Stripping Buffer was used following manufacturer's protocol (Thermo Scientific). Immunoreactive bands were visualized by ECL (Thermo, USA) according to the manufacturer’s instructions. The bands were revealed using Uvitec (UK) machine and digital images were collected. The relative densities of the immunoreactive bands were determined and normalized with respect to actin, using ImageJ software. Values were given as relative units (RU).

***Quantitative real-time PCR***

For gene expression analysis, the following protocol was used: total RNA was extracted by Trizol reagent, according to the manufacturer's instructions. The total RNA concentration was determined spectrophotometrically in RNAase-free water, and 1 µg aliquots of total RNA were reverse transcribed into cDNA using ProtoScript First Strand cDNA Synthesis Kit (NEB). RT-PCR was carried out on ABI 7300HT sequence detection system (ABI), in a total volume of 20µl containing EagleTaq Universal Master Mix (Roque), DEPC water, 4 µl of cDNA and the following the Prime Time qPCR Assays for CXCL1 Mm04207460m1 (Applied Biosystem, USA), TRVP4 ID qRnoCIP0027857 and TRPV1 ID qRnoCIP0024978 were purchased from Biorad (Biorad Laboratories; USA). Triplicate samples were run for each gene. The reference gene GADPH ID qRnoCIP0050838 (Biorad, USA) was used as an internal control to normalize the expression of target genes. RT-PCR protocol was: a pre-heating step for 3 min at 95 °C, 40 cycles at 95 °C for 10 s and 60° for 30 s and last end-step at 65 °C for 10 s. Relative expression levels were calculated for each sample after normalization against reference gene, using the ΔΔCt method for comparing relative fold expression differences, as previously described(51).

***Electrophysiological recording***

DRG-derived neurons were cultured for 7 days with the following combinations: paclitaxel (10 nM), paclitaxel + DF3966A (1µM final concentration), C5a (100 nM) and C5a + DF3966A. The compounds were administered for 1 min 30 sec (short acute stimulation) or 5 minutes (prolonged chronic stimulation). A wash-out followed the application of the compounds in the physiological control solution for an equivalent time (about 10 min). Electrophysiological recordings were performed by the patch-clamp technique in the whole-cell configuration. The standard extracellular solution was bath applied and contained the following (mM): NaCl 135, KCl 2, CaCl2 2, MgCl2 2, HEPES 10, glucose 5, pH 7.4. The standard pipet solution contained the following (mM): potassium aspartate 130, NaCl 10, MgCl2 2, CaCl2 1.3, EGTA 10, Hepes 10, pH 7.3. Recordings were acquired by the pClamp8.2 software and the MultiClamp 700A amplifier (Axon Instruments,CA, USA), in current-clamp mode.

***Proteome profiler assay***

M0 (differentiated, not stimulated) RAW 264.7 and THP-1 cells were treated with C5a (10 nM, diluted in medium) or paclitaxel (10 nM, diluted in medium), or DF3966A (1µM final concentration) or Avacopan (1µM) or C5aR1 antagonist (1µM) for 24 hours, then cells were gently washed with sterile PBS and the media was replaced with culture media (5ml) for 24h and then collected. For the control conditions, the cells were maintained in the culture media for 24, then the media was replaced with fresh media (5ml) for 24h and finally collected. Conditioned media were centrifugated to remove particulates and assayed immediately.

Sample amount was adjusted as suggested (500 µl). The reagents were prepared following manufacturer’s protocols (R&D, USA). Briefly, the membranes were incubated with Array Buffer 6 for 1 hour on a rocking platform shaker. The samples were prepared by adding up to 1 ml of Array Buffer 4 in two separated tubes and then 15 µl of reconstituted mouse cytokine detection antibody cocktail (> 40 mouse cytokines) to each prepared sample and incubated 1 hour. Then, the Array Buffer 6 was aspirated and replaced with the sample/antibody mixtures and incubated at 4˚ C overnight. The following day, membranes were washed 3 times and Streptavidin-HRP (1:2000) was incubated for 30 minutes at room temperature on a rocking platform shaker. The membranes were washed again, and 1 ml of prepared Chemi Reagent mix was placed onto each membrane. Multiple exposure times were acquired using UVITEC digital analyzer (Alliance, Cambridge, UK). The positive signals seen in the developed membranes can be identified by placing the transparency overlay template on the array image and aligning it with pairs of reference spots in the three corners of each array. Reference spots are included to demonstrate that the array has been incubated with Streptavidin-HRP during the assay procedure. Pixel densities (average signals of pair of duplicates which represent each cytokine) were analyzed by ImageJ and the average background was subtracted from each spot.

For the Human macrophages human XL Cytokine Array kit #Ary022B was used, while for murine macrophages mouse cytokine Array Panel A #Ary006 was used (R&D systems, USA).

***Animals***

Procedures involving animals and their care were conducted in conformity with International and National law and policies (EU Directive 2010/63/EU for animal experiments, ARRIVE guidelines(52), and the Basel declaration including the 3R conception). All behavioral tests were performed between 9:00 am and 1:00 pm, and mice were only used once. All efforts were made to minimize animal suffering. At the end of all experiments, the animals were sacrificed by cervical dislocation. The behavioral studies were performed on male Balb/C mice (6-8 weeks, Charles River) housed in the animal care facility of the Department of Pharmacy, University of Naples.

*C5ar*1^-/-^ (Jax mice stock number 006845) and control (BALB/cJ) male mice were used (PubMed: 8779720). *C5ar*1^-/-^ mice colony was maintained in the animal care facility of the Ribeirao Preto Medical School of Ribeirao Preto, University of Sao Paulo. Ethics in Animal Research of the Ribeirao Preto Medical School -USP. Animals were housed in a room with controlled temperature (22±1 °C), humidity (60±10%), and light (12 h per day); food and water were available ad libitum. Experiments on mice were performed one time and were specifically designed to use the minimum number of animals that was necessary to achieve the scientific objectives and to avoid performing unnecessary repetitions of experiments. In line with the 3 Rs principle (Replace, Reduce, Refine) of the Ethical Guidelines for the Use of Animals in Research, we calculated and selected the appropriate sample size in order to potentially obtain a statistical significance using a statistical power analysis program (G*Power software). Mice were randomly assigned to the different treatment groups. The investigator was blinded to the animal group allocation during the experiment and when assessing the outcome. All mice were included in the analysis.

***Drug treatments***

For CIPN model, paclitaxel solution (Onatax®, 100 mg/16.7 ml; cod 018155) was kindly provided by Prof. Thiago M. Cunha (Department of Pharmacology, Ribeirao Preto Medical School, University of Sao Paulo, Brazil). For the *in vivo* experiments, DF3966A (Dompé farmaceutici S.p.A., L'Aquila, Italy) was dissolved in water (30 mg/10 ml), and orally given at a dose of 30 mg/kg every 12 hours for 14 days during the induction phase of CIPN. On days 1, 3, 5, and 7, DF3966A was administrated 1 hour after paclitaxel.

For HSR model, paclitaxel (Selleckchem, USA, cod. S1150) was intravenous administrated at 20 mg/kg (0.1mL/Kg) dissolved in Cremophor EL (polyoxyl 35 castor oil, Sigma-Aldrich, cod. 238470) or in PEG400/TWEEN80 (Sigma-Aldrich). Vehicle DF3966A (saline) and DF3966A (30mg/kg, os) were administrated 3h before paclitaxel, while vehicle Avacopan (PEG-400/solutol-HS15 70:30, 5 mL/kg(29)) and Avacopan (30 mg/kg, os) were administrated 1h before paclitaxel.

***Induction of peripheral neuropathy by paclitaxel***

The protocol used was performed according to Polomano et al., 2001(19) and adapted to mice. Paclitaxel solution (100 mg/ 16.7 ml) was diluted with saline to obtain a dose of 4 mg/kg; animals received intraperitoneally (i.p.) 0.3 ml of this paclitaxel solution for four alternate days (1, 3, 5, and 7). During treatment period, animals were measured 4 hours after the injection of paclitaxel.

***Mechanical allodynia***

To assess changes in sensitization or in the development of mechanical allodynia, sensitivity to tactile stimulation was measured using the Dynamic Plantar Aesthesiometer (DPA, Ugo Basile, Italy). Animals were placed in a chamber with a mesh metal floor covered by a plastic dome that enables the animals to walk freely, but not to jump, in a quiet room 15-30 minutes before testing. The mechanical stimulus was then delivered in the mid-plantar skin of the hind paw. The DPA automatically records the force at which the foot is withdrawn. The cut-off was fixed at 5 g. Testing was performed 3h after DF3966A treatment on days 1-3-5-7-10-14.

In C5ar1^-/-^ mice and relative WT group, mechanical allodynia was tested using von Frey filaments. A crescent series of filaments were applied on the right paw of mice. The lower filament able to elicit flinching movements was recorded as the mechanical threshold. The results are expressed as a log of mechanical threshold. During treatment period, animals were measured 4 hours after the injection of paclitaxel.

***Cold allodynia***

Cold sensitivity was measured as the number of foot withdrawal responses after the application of acetone to the dorsal surface of the paw(10). A drop (50 µL) of acetone was applied to the dorsal surface of paws with a syringe connected to a thin polyethylene tube. A brisk foot withdrawal response (licking, flinching or lifting) after the spread of acetone over the dorsal surface of the paw was considered as a sign of cold allodynia. Cold responses were measured 3h after DF3966A treatment on days 1, 3, 5, 7, 10, 14.

In C5ar1^-/-^ mice and relative WT group, after the application of acetone, withdrawal response was evaluated with a chronometer in a total testing time of 2 minutes. The animals were tested nearly 15 minutes after the mechanical test.

***Real-time PCR analysis on paws and spinal cord***

For this experiment, we used paws and spinal cord samples collected at day 15. Total RNA, isolated from paws and spinal cord, was extracted using TRIzol Reagent (Bio-Rad Laboratories), according to the manufacturer’s instructions. cDNA was synthesized using a reverse transcription kit (Maxima First Strand cDNA Synthesized Kit, Fermentas, Ontario, Canada) from 2 µg total RNA. PCRs were performed with a Bio-Rad CFX96 Connect Real-time PCR System instrument and software (Bio-Rad Laboratories). The PCR conditions were 15 min at 95 °C followed by 40 cycles of two-step PCR denaturation at 94 °C for 15 s, annealing at 55 °C for 30 s and extension at 72 °C for 30 s. Each sample contained 20 ng cDNA in 2X QuantiTect SYBRGreen PCR Master Mix and primers pairs to amplify complement component 5a receptor (c5aR1; Cat.n° QT00288232), transient receptor potential cation channel subfamily V member 1 (TRPV1; Qiagen, Hilden, Germany ID QT00167048), TNF-α (Tnf; Qiagen, Hilden, Germany, ID QT00104006), interleukin-1β (Il1β; Qiagen, Hilden, Germany, ID QT01048355) and interleukin-6 (IL6; Qiagen, Hilden, Germany, ID QT00098875) in a final volume of 50 μl. The relative amount of each studied mRNA was normalized to β-Actin as refence gene, and data were analyzed according to the 2^−ΔΔCT^ method(51).

***Assessment of Vascular Leakage***

Evans Blue (EB, 0.8%/0.3mL/iv) was used to visualize the ear blue colour and quantify the extent of vascular leakage in ears, so as to assess the skin manifestation of paclitaxel injection induced HSRs. 30 minutes after paclitaxel administration mice received EB; the degree of ear colouring was assessed by a score from 0 - 5 as described(53), where "0" did not represent the visible blue area and "1 to 5" indicated the ratio of the visible blue area.

***Plasma extravasation***

About 40 minutes following paclitaxel administration, the animal was sacrificed, the ears were removed, shredded and stored in 2mL of formamide, and incubated overnight at 55 °C. The day after the ears were centrifuged and the supernatant was taken and analysed by the spectrophotometer (620 nm O.D.). Formamide, in duplicate, was used as blank. Results are expressed as μg per mg of tissue.

***ELISA assay***

Intracardiac injection was done to obtained blood in tubes and leaving it undisturbed at room temperature. Serum was separated from blood cells 30 minutes later by centrifugation (2000 x g for 10 min, at 4°C). Aliquots were prepared and stored at -20°C. ELISA kit was used to evaluate the following marker: histamine and SC5b-9 (Mybiosource, San Diego, USA Cat.No: MBS9361379, MBS725193).

***Data analysis***

All data are presented as the mean ± SEM. Analysis of data was conducted using GraphPad Prism 8 (GraphPad Software Inc., San Diego, CA, USA). The significance of differences between groups *in vivo* behavioral testing experiments was determined on basis of test used, by one or two-way analyses of variance (ANOVA), followed by Bonferroni or by Tukey post hoc tests for multiple comparisons as appropriate. Since no significant difference was evaluated between sham vehicle-mice and sham DF3966A-mice we report only one sham group. The level of significance was set at *P < 0.05.

The significance of differences between groups *in vivo* HSR testing experiments was determined on basis of test used, by one-way ANOVA followed by Dunnett’s test for multiple comparisons. Regarding the *in vitro* experiments, one-way ANOVA followed by Tukey’s post-hoc test was performed. No samples or animals were excluded from the analysis.

**Conflict of Interest**

Brandolini L, Novelli R, Giorgio C, Sirico S, Cocchiaro P, Ruocco A, Amendola P, Talarico C, Manelfi C, Iaconis D, Beccari A, Aramini A and Allegretti M are employees of Dompé Farmaceutici S.p.A., Italy. The other authors declare no conflict of interest.

**Data Availability Statement**

The datasets used and/or analyzed during the current study are available from the corresponding author on reasonable request.

**Funding**

This work was supported by the grants “Industria Sostenibile - PON I&C 2014/2020 D.M. 1 giugno 2016 F/090033/01-03-04/X36” and “Sviluppo di Approcci Terapeutici INnovativi per patologie neoplastiche resistenti ai trattamenti - Satin - POR FESR Campania 2014‐2020”.

**References**

1. Sadeghi-aliabadi H, Yegdaneh A. Taxol and Related Compounds. In: Ramawat KG, Mérillon JM, editors. Natural Products: Phytochemistry, Botany and Metabolism of Alkaloids, Phenolics and Terpenes [Internet]. Berlin, Heidelberg: Springer; 2013 [cited 2021 May 18]. p. 3159–71. Available from: https://doi.org/10.1007/978-3-642-22144-6_143

2. Barbuti AM, Chen ZS. Paclitaxel Through the Ages of Anticancer Therapy: Exploring Its Role in Chemoresistance and Radiation Therapy. Cancers. 2015 Dec 3;7(4):2360–71.

3. Chou PL, Huang YP, Cheng MH, Rau KM, Fang YP. Improvement of Paclitaxel-Associated Adverse Reactions (ADRs) via the Use of Nano-Based Drug Delivery Systems: A Systematic Review and Network Meta-Analysis. Int J Nanomedicine. 2020 Mar 12;15:1731–43.

4. Boulanger J, Boursiquot JN, Cournoyer G, Lemieux J, Masse MS, Almanric K, et al. Management of hypersensitivity to platinum- and taxane-based chemotherapy: cepo review and clinical recommendations. Curr Oncol. 2014 Aug;21(4):e630–41.

5. Guitart MC. Rapid Drug Desensitization for Hypersensitivity Reactions to Chemotherapy and Monoclonal Antibodies in the 21st Century. J Investig Allergol Clin Immunol. 2014;24:8.

6. Staff NP, Fehrenbacher JC, Caillaud M, Damaj MI, Segal RA, Rieger S. Pathogenesis of paclitaxel-induced peripheral neuropathy: A current review of in vitro and in vivo findings using rodent and human model systems. Exp Neurol. 2020 Feb;324:113121.

7. Zajączkowska R, Kocot-Kępska M, Leppert W, Wrzosek A, Mika J, Wordliczek J. Mechanisms of Chemotherapy-Induced Peripheral Neuropathy. Int J Mol Sci [Internet]. 2019 Mar 22 [cited 2021 May 18];20(6). Available from: https://www.ncbi.nlm.nih.gov/pmc/articles/PMC6471666/

8. Son S, Shim DW, Hwang I, Park JH, Yu JW. Chemotherapeutic Agent Paclitaxel Mediates Priming of NLRP3 Inflammasome Activation. Front Immunol. 2019;10:1108.

9. Collins TS, Lee LF, Ting JP. Paclitaxel up-regulates interleukin-8 synthesis in human lung carcinoma through an NF-kappaB- and AP-1-dependent mechanism. Cancer Immunol Immunother CII. 2000 May;49(2):78–84.

10. Brandolini L, Elisabetta B, Adelchi RP, Roberto R, Loredana C, Andrea A, et al. CXCR1/2 pathways in paclitaxel-induced neuropathic pain. Oncotarget. 2017 Feb 20;8(14):23188–201.

11. Brandolini L, Castelli V, Aramini A, Giorgio C, Bianchini G, Russo R, et al. DF2726A, a new IL-8 signalling inhibitor, is able to counteract chemotherapy-induced neuropathic pain. Sci Rep. 2019 Aug 13;9(1):11729.

12. Giorgio C, Zippoli M, Cocchiaro P, Castelli V, Varrassi G, Aramini A, et al. Emerging Role of C5 Complement Pathway in Peripheral Neuropathies: Current Treatments and Future Perspectives. Biomedicines. 2021 Apr;9(4):399.

13. Rs G, M C, Gj B, Ch M, J S, A M, et al. Complement induction in spinal cord microglia results in anaphylatoxin C5a-mediated pain hypersensitivity. J Neurosci Off J Soc Neurosci. 2007 Aug 1;27(32):8699–708.

14. Brandolini L, Grannonico M, Bianchini G, Colanardi A, Sebastiani P, Paladini A, et al. The Novel C5aR Antagonist DF3016A Protects Neurons Against Ischemic Neuroinflammatory Injury. Neurotox Res. 2019 Jul;36(1):163–74.

15. Beato C, Beccari AR, Cavazzoni C, Lorenzi S, Costantino G. Use of Experimental Design To Optimize Docking Performance: The Case of LiGenDock, the Docking Module of Ligen, a New De Novo Design Program. J Chem Inf Model. 2013 Jun 24;53(6):1503–17.

16. Bertini R, Barcelos LS, Beccari AR, Cavalieri B, Moriconi A, Bizzarri C, et al. Receptor binding mode and pharmacological characterization of a potent and selective dual CXCR1/CXCR2 non-competitive allosteric inhibitor. Br J Pharmacol. 2012 Jan;165(2):436–54.

17. Olaru A, Bala C, Jaffrezic-Renault N, Aboul-Enein HY. Surface Plasmon Resonance (SPR) Biosensors in Pharmaceutical Analysis. Crit Rev Anal Chem. 2015 Apr 3;45(2):97–105.

18. Yassine F, Salibi E, Gali-Muhtasib H. Overview of the Formulations and Analogs in the Taxanes’ Story. Curr Med Chem. 2016;23(40):4540–58.

19. Polomano RC, Mannes AJ, Clark US, Bennett GJ. A painful peripheral neuropathy in the rat produced by the chemotherapeutic drug, paclitaxel. Pain. 2001 Dec;94(3):293–304.

20. Svensson CI, Schäfers M, Jones TL, Powell H, Sorkin LS. Spinal blockade of TNF blocks spinal nerve ligation-induced increases in spinal P-p38. Neurosci Lett. 2005 May 13;379(3):209–13.

21. Koehl J, Kordowski A, Wu D, Hogan SP. Experimental food-induced anaphylaxis is driven by C5aR1 actvivation on mast cells. J Immunol. 2017 May 1;198(1 Supplement):145.2-145.2.

22. Töro K, Borka K, Kardos M, Kristóf I, Sótonyi P. Expression and function of C5a receptor in a fatal anaphylaxis after honey bee sting. J Forensic Sci. 2011 Mar;56(2):526–8.

23. Tsubota M, Fukuda R, Hayashi Y, Miyazaki T, Ueda S, Yamashita R, et al. Role of non-macrophage cell-derived HMGB1 in oxaliplatin-induced peripheral neuropathy and its prevention by the thrombin/thrombomodulin system in rodents: negative impact of anticoagulants. J Neuroinflammation. 2019 Oct 30;16(1):199.

24. Escribese MM, Rosace D, Chivato T, Fernández TD, Corbí AL, Barber D. Alternative Anaphylactic Routes: The Potential Role of Macrophages. Front Immunol. 2017 May 8;8:515.

25. Tang H, Amara U, Tang D, Barnes MA, McDonald C, Nagy LE. Synergistic interaction between C5a and NOD2 signaling in the regulation of chemokine expression in RAW 264.7 macrophages. Adv Biosci Biotechnol Print. 2013 Aug;4(8C):30–7.

26. Lei YC, Lu CL, Chen L, Ge K, Yang LL, Li W, et al. C5a/C5aR pathway is essential for up-regulating SphK1 expression through p38-MAPK activation in acute liver failure. World J Gastroenterol. 2016 Dec 14;22(46):10148–57.

27. Korosec P, Turner PJ, Silar M, Kopac P, Kosnik M, Gibbs BF, et al. Basophils, high-affinity IgE receptors, and CCL2 in human anaphylaxis. J Allergy Clin Immunol. 2017 Sep;140(3):750-758.e15.

28. Jayne DRW, Bruchfeld AN, Harper L, Schaier M, Venning MC, Hamilton P, et al. Randomized Trial of C5a Receptor Inhibitor Avacopan in ANCA-Associated Vasculitis. J Am Soc Nephrol JASN. 2017 Sep;28(9):2756–67.

29. Bekker P, Dairaghi D, Seitz L, Leleti M, Wang Y, Ertl L, et al. Characterization of Pharmacologic and Pharmacokinetic Properties of CCX168, a Potent and Selective Orally Administered Complement 5a Receptor Inhibitor, Based on Preclinical Evaluation and Randomized Phase 1 Clinical Study. PloS One. 2016;11(10):e0164646.

30. Allegretti M, Cesta MC, Zippoli M, Beccari A, Talarico C, Mantelli F, et al. Repurposing the estrogen receptor modulator raloxifene to treat SARS-CoV-2 infection. Cell Death Differ. 2021 Aug 17;1–11.

31. Takahashi A, de Andrés MC, Hashimoto K, Itoi E, Oreffo ROC. Epigenetic regulation of interleukin-8, an inflammatory chemokine, in osteoarthritis. Osteoarthritis Cartilage. 2015 Nov;23(11):1946–54.

32. Fritzinger DC, Benjamin DE. The Complement System in Neuropathic and Postoperative Pain. Open Pain J. 2016;9:26–37.

33. Xu J, Zhang L, Xie M, Li Y, Huang P, Saunders TL, et al. Role of Complement in a Rat Model of Paclitaxel-Induced Peripheral Neuropathy. J Immunol Baltim Md 1950. 2018 Jun 15;200(12):4094–101.

34. Leal AD, Qin R, Atherton PJ, Haluska P, Behrens RJ, Tiber C, et al. NCCTG N08CA (Alliance): The use of Glutathione for Prevention of Paclitaxel/Carboplatin Induced Peripheral Neuropathy: A Phase III Randomized, Double-Blind Placebo-Controlled Study. Cancer. 2014 Jun 15;120(12):1890–7.

35. Nishise S, Takeda Y, Nishise Y, Fujishima S, Orii T, Otake S, et al. Biological effect of anaphylatoxin C5a on the generation of anti-inflammatory substances in leukocyte adsorption. Ther Apher Dial Off Peer-Rev J Int Soc Apher Jpn Soc Apher Jpn Soc Dial Ther. 2009 Dec;13(6):509–14.

36. Laumonnier Y, Wiese AV, Figge J, Karsten C. Regulation and function of anaphylatoxins and their receptors in allergic asthma. Mol Immunol. 2017 Apr 1;84:51–6.

37. Arbore G, West EE, Spolski R, Robertson AAB, Klos A, Rheinheimer C, et al. T helper 1 immunity requires complement-driven NLRP3 inflammasome activity in CD4+ T cells. Science. 2016 Jun 17;352(6292):aad1210.

38. Zeng Q zhen, Yang F, Li C guang, Xu L hui, He X hui, Mai F yi, et al. Paclitaxel Enhances the Innate Immunity by Promoting NLRP3 Inflammasome Activation in Macrophages. Front Immunol [Internet]. 2019 [cited 2022 Apr 5];10. Available from: https://www.frontiersin.org/article/10.3389/fimmu.2019.00072

39. Peng L, Bu Z, Ye X, Zhou Y, Zhao Q. Incidence and risk of peripheral neuropathy with nab-paclitaxel in patients with cancer: a meta-analysis. Eur J Cancer Care (Engl). 2017 Sep;26(5).

40. Guo X, Sun H, Dong J, Feng Y, Li H, Zhuang R, et al. Does nab-paclitaxel have a higher incidence of peripheral neuropathy than solvent-based paclitaxel? Evidence from a systematic review and meta-analysis. Crit Rev Oncol Hematol. 2019 Jul 1;139:16–23.

41. Picard M, Castells MC. Re-visiting Hypersensitivity Reactions to Taxanes: A Comprehensive Review. Clin Rev Allergy Immunol. 2015 Oct;49(2):177–91.

42. Ding P, Li L, Li L, Lv X, Zhou D, Wang Q, et al. C5aR1 is a master regulator in Colorectal Tumorigenesis via Immune modulation. Theranostics. 2020;10(19):8619–32.

43. Ajona D, Ortiz-Espinosa S, Moreno H, Lozano T, Pajares MJ, Agorreta J, et al. A Combined PD-1/C5a Blockade Synergistically Protects against Lung Cancer Growth and Metastasis. Cancer Discov. 2017 Jul;7(7):694–703.

44. Thurman JM, Laskowski J, Nemenoff RA. Complement and Cancer—A Dysfunctional Relationship? Antibodies. 2020 Nov 5;9(4):61.

45. Schott AF, Goldstein LJ, Cristofanilli M, Ruffini PA, McCanna S, Reuben JM, et al. Phase Ib Pilot Study to Evaluate Reparixin in Combination with Weekly Paclitaxel in Patients with HER-2–Negative Metastatic Breast Cancer. Clin Cancer Res. 2017 Sep 15;23(18):5358–65.

46. Vanneman M, Dranoff G. Combining Immunotherapy and Targeted Therapies in Cancer Treatment. Nat Rev Cancer. 2012 Mar 22;12(4):237–51.

47. Nakajima TE, Kadowaki S, Minashi K, Nishina T, Yamanaka T, Hayashi Y, et al. Multicenter Phase I/II Study of Nivolumab Combined with Paclitaxel Plus Ramucirumab as Second-line Treatment in Patients with Advanced Gastric Cancer. Clin Cancer Res. 2021 Feb 15;27(4):1029–36.

48. Kloover JS, Bakker MA den, Gelderblom H, Meerbeeck JP van. Fatal outcome of a hypersensitivity reaction to paclitaxel: a critical review of premedication regimens. Br J Cancer. 2004 Jan 26;90(2):304–5.

49. O’Meara RW, Ryan SD, Colognato H, Kothary R. Derivation of enriched oligodendrocyte cultures and oligodendrocyte/neuron myelinating co-cultures from post-natal murine tissues. J Vis Exp JoVE. 2011 Aug 21;(54):3324.

50. Owen DE, Egerton J. Culture of Dissociated Sensory Neurons from Dorsal Root Ganglia of Postnatal and Adult Rats. In: Skaper SD, editor. Neurotrophic Factors: Methods and Protocols [Internet]. Totowa, NJ: Humana Press; 2012 [cited 2021 Dec 13]. p. 179–87. (Methods in Molecular Biology). Available from: https://doi.org/10.1007/978-1-61779-536-7_16

51. Livak KJ, Schmittgen TD. Analysis of relative gene expression data using real-time quantitative PCR and the 2(-Delta Delta C(T)) Method. Methods San Diego Calif. 2001 Dec;25(4):402–8.

52. Sert NP du, Hurst V, Ahluwalia A, Alam S, Avey MT, Baker M, et al. The ARRIVE guidelines 2.0: Updated guidelines for reporting animal research. PLOS Biol. 2020 Jul 14;18(7):e3000410.

53. Pan P, Zhang Q, Liu W, Wang W, Lao Z, Zhang W, et al. Dengue Virus M Protein Promotes NLRP3 Inflammasome Activation To Induce Vascular Leakage in Mice. J Virol. 2019 Oct 15;93(21):e00996-19.

**Table 1.** Selected potential paclitaxel targets and their sources with the corresponding *in silico* classification based on docking experiments. n.b. are non-bound protein.

| **Target** | **Predicted Affinity** |
| --- | --- |
| TLR2_HUMAN | High |
| NK1R_HUMAN | High |
| EDNRB_HUMAN | High |
| EGFR_HUMAN | High |
| C5AR1_HUMAN | High |
| EDNRA_HUMAN | High |
| PAR2_HUMAN | Low |
| C5AR2_HUMAN | Low |
| AA2BR_HUMAN | Low |
| RET_HUMAN | Low |
| CRFR2_HUMAN | Low |
| TNR1A_HUMAN | Low |
| S1PR1_HUMAN | n.b |
| PAR1_HUMAN | n.b |
| KIT_HUMAN | n.b |
| ADRB2_HUMAN | n.b |
| TLR8_HUMAN | n.b |
| LPAR1_HUMAN | n.b |
| TGFR2_HUMAN | n.b |
| DDR1_HUMAN | n.b |
| NTR1_HUMAN | n.b |
| PTAFR_HUMAN | n.b |
| C3AR_HUMAN | n.b |
| PE2R2_HUMAN | n.b |
| ACM3_HUMAN | n.b |
| PE2R3_HUMAN | n.b |
| PE2R4_HUMAN | n.b |

**FIGURE LEGENDS**

**Figure 1**. **Paclitaxel binding mode in C5aR1 binding site.** The ligand between C5aR1 and paclitaxel is reported in green lines, while the protein structure is reported in blue surface (A) and cartoon (B). The contact residues and related hydrogen bonds are depicted in slate sticks and yellow dots, respectively.

**Figure 2.** **Paclitaxel and C5aR1 binding studies by SPR.** A) Binding of paclitaxel on immobilized C5aR1, C5aR2, C5a and C5a des-Arg. B) Dose-dependent binding of paclitaxel on immobilized C5aR1. C and D) Co-injection of paclitaxel and C5aR1 on immobilized C5a and relative IC50. E and F) Co-injection of paclitaxel and C5aR1 on immobilized C5a des-Arg and relative IC50. G and H) Co-injection of paclitaxel and C5aR1 on immobilized monoclonal antibody anti-C5aR1 and relative IC50.

**Figure 3. Paclitaxel-C5aR1 intracellular pathway.** Quantification of the expression of phosphorylated and total forms of NFκB (A), c-Jun (B), c-Fos (C), p38 MAPK (D) and ERK3/MAPK6 (E) in differentiated F11 cells following paclitaxel (PAC) treatment evaluated by western blot analysis (F). F11 cells were treated for 24 hours with PAC (10nM) and DF3966A (10µM). ***P<0.0005; *P<0.05 vs CTR; ^+++^P<0.0005 vs paclitaxel. Data are mean ± SEM of n=3 different experiments.

**Figure 4. C5aR1 inhibition restores neuronal plasticity in paclitaxel-treated F11 cells.** Quantification of the expression of phosphorylated and total forms of p-cortactin (A), p-FAK (B), p-JAK2 (C) and p-STAT3 (D) in differentiated F11 cells following paclitaxel (PAC) or C5a treatment evaluated by western blot analysis. Expression of p-cortactin and p-FAK (E), p-JAK2 and p-STAT3 (F), and TRPV1 and TRPV4 (G) in differentiated F11 cells exposed to paclitaxel and treated or not with a C5aR1 inhibitor, DF3966A. F11 cells were treated for 24 hours with PAC (10nM), C5a (50nM) or DF3966A (10µM) or PAC (10nM) + DF3966A (10µM). ***P<0.0005; **P<0.005; *P<0.05 vs CTR; +++P<0.0005; ++ P<0.005; +P<0.05 vs paclitaxel. Data are mean ± SEM of n=3 different experiments.

**Figure 5. C5aR1 inhibition counteracts paclitaxel-induced alterations in rodent DRG.** Representative trace of electrophysiological recordings in DRG cells maintained under basal conditions (A), and in cells challenged with paclitaxel (10 nM) (PAC) (B), paclitaxel+DF3966A (1µM) (C), C5a (100 nM) (D) or C5a+DF3966A (E). Quantitative evaluation of action potential firing rate in DRG exposed to paclitaxel alone or in combination with DF3966A (F), and C5a alone or in combination with DF3966A (G). DRG-derived neurons were cultured for 7 days. Cells were treated for 1 min 30 sec (short) or 5 minutes (long). **P<0.005 and *P<0.05 vs respective basal condition; +P<0.05 vs paclitaxel or C5a. Data are mean ± SEM of n=3 different experiments.

**Figure 6.** **C5aR1 is a key mediator of paclitaxel-induced cold and mechanical allodynia.** Time course of paw withdrawal responses in mice displaying paclitaxel (PAC)-induced cold (A) (two-way ANOVA, effect of time x treatment F (10,285)=21.69; p<0.0001 followed by Bonferroni’s multiple comparisons test) and mechanical (B) (two-way ANOVA, effect of time x treatment F (10,285)=17.63; p<0.0001 followed by Bonferroni’s multiple comparisons test) allodynia treated or not with a C5aR1 inhibitor, DF3966A (30 mg/kg). DF3966A was orally administered every 12 hours for 14 days during the induction phase of CIPN. Cold and mechanical allodynia were evaluated 3h after DF3966A treatment on days 1, 3, 5, 7, 10 and 14. Real time-PCR analysis of C5aR1 (one-way ANOVA, effect of treatment F (2,27)=6.381; p=0.0054 followed by Bonferroni’s multiple comparisons test) and TNF-α (one-way ANOVA, effect of treatment F (2,27)=14.79; p<0.0001 followed by Bonferroni’s multiple comparisons test) expression levels in the spinal cord, and of C5aR1 (one-way ANOVA, effect of treatment F (2,27)=3.480; p=0.0452 followed by Bonferroni’s multiple comparisons test), TRPV1 (one-way ANOVA, effect of treatment F (2,27)=6.349; p=0.0055 followed by Bonferroni’s multiple comparisons test), TRPV4 (one-way ANOVA, effect of treatment F (2,27)=4.050; p=0.0290 followed by Bonferroni’s multiple comparisons test), IL-1β (one-way ANOVA, effect of treatment F (2,27)=13.81; p<0.0001 followed by Bonferroni’s multiple comparisons test) and IL-6 (one-way ANOVA, effect of treatment F (2,27)=1.898; p=0.1694 followed by Bonferroni’s multiple comparisons test) in paw samples collected at day 15 after the first paclitaxel administration. ***P<0.0005 and *P<0.05 vs respective sham group; ; ^+++^P<0.0005; ^++^P<0.005 and ^+^P<0.05 vs paclitaxel. Data are expressed as mean ± SEM; n=10 per group.

**Figure 7.** **C5aR1 inhibition blocks C5a- and paclitaxel-induced anaphylactoid cytokine release from mouse and human macrophages.** Proteome profiles of cytokines released from RAW 264.7 (A and B) and human (C and D) macrophages challenged with C5a (A and C) or paclitaxel (B and D) alone or in combination with a C5AR1 antibody (anti C5AR), Avacopan or DF3966A. RAW 264.7 and human macrophages cells were treated with C5a (10 nM), or paclitaxel (PAC) (10 nM), or DF3966A (1μM) or Avacopan (1μM) and C5aR1 antibody (1μM)) for 24 hours. Data are mean ± SEM of n=3 different experiments.

**Figure 8. C5aR1 inhibition prevents anaphylactoid reactions induced by paclitaxel in mice.** Effect of vehicle, DF3966A or Avacopan on paclitaxel-increased vascular permeability (A) (one-way ANOVA, effect of treatment F (4,44)=5.121; p=0.0018 followed by Bonferroni’s multiple comparisons test) and plasma extravasation (B) (one-way ANOVA, effect of treatment F (4,44)=7.258; p=0.0001 followed by Bonferroni’s multiple comparisons test) in mice. Serum histamine (one-way ANOVA, effect of treatment F (3,36)=7.590; p=0.0005 followed by Bonferroni’s multiple comparisons test) (C) and SC5B-9 (one-way ANOVA, effect of treatment F (3,36)=24.74; p<0.0001 followed by Bonferroni’s multiple comparisons test) (D) concentration in mice receiving paclitaxel pre-treated with vehicle, DF3966A or Avacopan. Paclitaxel was intravenously administered at 20 mg/kg. Vehicle DF3966A (saline) and DF3966A (30mg/kg, os) were administered 3h before paclitaxel, while vehicle Avacopan and Avacopan (30 mg/kg, os) were administered 1h before paclitaxel. ***P<0.0005, **P<0.005 and *P<0.05 vs Cremophor group; ^+++^P<0.0005; ^++^P<0.005 and ^+^P<0.05 vs paclitaxel. Data are shown as mean ± SEM of 10 animals per group.
