# Supplementary material for: Paclitaxel binds and activates C5aR1: A new potential therapeutic target for the prevention of chemotherapy-induced peripheral neuropathy and hypersensitivity reactions
Source: Cell Death Dis. 2022 May 25;13(5):500. doi: 10.1038/s41419-022-04964-w (PMC9130998; doi:10.1038/s41419-022-04964-w)
Supplement: Supplementary file 5 [file 41419_2022_4964_MOESM1_ESM.odt]

Supplementary Information and Figures

Paclitaxel binds and activates C5aR1: a new potential therapeutic target for the prevention of chemotherapy-induced peripheral neuropathy and hypersensitivity reactions

Brandolini Laura1*, d’Angelo Michele2*, Novelli Rubina3, Castelli Vanessa2, Giorgio Cristina4, Sirico Anna4, Cocchiaro Pasquale4, D’Egidio Francesco2, Benedetti Elisabetta2, Cristiano Claudia5, Bugatti Antonella 6, Ruocco Anna 4, Amendola Pier Giorgio4, Talarico Carmine4, Manelfi Candida 4, Iaconis Daniela 4, Beccari Andrea4, Quadros Andreza U7, Cunha Thiago M7, Caruso Arnaldo 6, Russo Roberto5, Cimini Annamaria2,8, Aramini Andrea1, Allegretti Marcello1

1Dompé Farmaceutici SpA, Via Campo di Pile, 67100 L’Aquila, Italy

2Department of Life, Health and Environmental Sciences, University of L’Aquila, 67100 L’Aquila Italy

3 Dompé Farmaceutici SpA, Via S. Lucia, 20122 Milan, Italy

4 Dompé Farmaceutici SpA, Via De Amicis, 80131 Naples, Italy

5 Department of Pharmacy, University of Naples Federico II, 80131 Naples, Italy

6 Department of Molecular and Traslational Medicine, University of Brescia Medical School, 25123 Brescia, Italy

7 Center for Research in Inflammatory Diseases (CRID), Department of Pharmacology, Ribeirao Preto Medical School, University of Sao Paulo, Ribeirão Preto - SP, 14049-900, Brazil

8Sbarro Institute for Cancer Research and Molecular Medicine and Center for Biotechnology, Temple University, Philadelphia, PA 19122, USA

* These authors contributed equally to this work

Running title: C5aR1 mediates paclitaxel neuropathy and anaphylaxis

Corresponding author

Marcello Allegretti

marcello.allegretti@dompe.com

Supplementary Information

- Selection of IL-8 upstream mediators as potential targets of paclitaxel

Starting from our data demonstrating the crucial role of the IL-8 pathway in the pathophysiology of paclitaxel-induced CIPN and the potential therapeutic application of IL-8 inhibition in this condition(1), we sought to investigate the upstream pathway that is activated by paclitaxel and can mediate IL-8 production. 

To this end, we first performed a bioinformatic analysis using MetaCore™, a Cortellis solution from Clarivate, which provides an integrated software suite for functional analysis based on a curated database of several information, such as human protein-protein, protein-DNA interactions, transcription factors, signaling and metabolic pathways. We queried the database with IL-8 and looked for incoming functional interactions; in particular, we looked for all the proteins that activate the expression of this chemokine. We obtained 38 proteins, classified as receptors: 7 are described as receptors with enzymatic activity and 31 as G-protein-coupled receptors (GPCRs). In parallel with the bioinformatic analysis, we also retrieved from the literature information on IL-8 upstream mediators that could be potential targets of paclitaxel; among them: C5aR1, which is reported to mediate C5a-induced IL-8 gene expression in various cell types and conditions(2–4); the interleukin-1 receptor (IL-1R), which activates downstream kinases finally inducing IL-8 gene expression(5–7); the tumor necrosis factor receptor 1 (TNFR1), which mediates TNF-α-induced IL-8 expression and secretion(5,7); the toll-like receptor 4 (TLR4), which induces the expression of IL-8 mRNA and protein synthesis through the activation of downstream signaling (e.g. the nuclear factor kappaB, NF-κB)(8); and the E-type prostanoid receptor 4 (EP4), which mediates prostaglandin E2 (PGE2)-induced IL-8 expression(9,10). Notably, some of the receptors that we selected from the literature search [i.e. anaphylatoxin chemotactic (C5aR) and prostaglandin E2 receptor families] overlapped with the bioinformatic results, confirming the goodness of this latter analysis. 

  - List of known targets of paclitaxel retrieved from GoStar database

| COMMON NAME |
| --- |
| VEGFR2 |
| UDP-GLUCURONOSYLTRANSFERASE 2B7 |
| UDP-GLUCURONOSYLTRANSFERASE 2B10 |
| UDP-GLUCURONOSYLTRANSFERASE 1A6 |
| UDP-GLUCURONOSYLTRANSFERASE 1A4 |
| UDP-GLUCURONOSYLTRANSFERASE 1A1 |
| TUMOR PROTEIN P53 |
| TUBULIN |
| RHODANESE |
| QUINONE REDUCTASE 1 |
| PROTEIN ARGININE DEIMINASE 4 |
| POLY [ADP-RIBOSE] POLYMERASE 1 |
| PKC IOTA |
| NFKB |
| MULTIDRUG RESISTANCE-ASSOCIATED PROTEIN 7 |
| MULTIDRUG RESISTANCE-ASSOCIATED PROTEIN |
| MULTIDRUG RESISTANCE PROTEIN 1 |
| MITOTIC KINESIN-LIKE PROTEIN 2 |
| MITOGEN-ACTIVATED PROTEIN KINASE |
| MALATE DEHYDROGENASE |
| HSP90 ALPHA |
| HSP60/10 (CHAPERONIN) |
| HSP60 |
| HELICASE |
| HDAC |
| GROEL-GROES CHAPERONIN |
| FGFR1 |
| FACILITATED GLUCOSE TRANSPORTER 1 |
| EGFR |
| DNA REPAIR PROTEIN RAD51 HOMOLOG 1 |
| C-SRC TYROSINE KINASE |
| COPPER-TRANSPORTING ATPASE 1 |
| C-MER PROTO-ONCOGENE TYROSINE KINASE |
| BRUTON'S TYROSINE KINASE |
| BETA TUBULIN |
| BETA III TUBULIN |
| BCR-ABL |
| ARGININE KINASE |
| ALPHA GLUCOSIDASE |
| ABL1 |
| ABC TRANSPORTER G2 |

- Docetaxel SPR studies 

We performed SPR experiments to investigate whether docetaxel could interfere with the interaction between C5aR1 and C5aR2 and their natural ligands (C5a and C5a des-Arg). Experiments showed that docetaxel can bind the immobilized C5aR1 receptor in a dose dependent way with an affinity value of 9.752 µM (28.48 µM at equilibrium) (Figure S2). On the other hand and similarly to paclitaxel, docetaxel did not bind to immobilized C5aR2 receptor, and neither to C5a or C5a des-Arg proteins immobilized onto a sensor chip. Finally, co-injection experiments, in which C5aR1 receptor was injected on different surfaces (C5a and C5a des-Arg) in the absence or presence of different concentrations of docetaxel, showed that docetaxel could inhibit both C5aR1/C5a and C5aR1/C5a des-Arg interactions in a dose-dependent way, with IC50 values of 16.15 and 73.15 μM, respectively (Figure S2). By co-injection experiments with CD88 (C5aR1 neutralizing antibody), we also demonstrated that docetaxel acts in orthosteric mode on C5aR1 receptor, as it inhibited C5aR1/CD88 antibody interaction in a dose-dependent way with an IC50 value of 21.04 μM.

- In vitro investigation of paclitaxel-C5aR1 intracellular pathway

To investigate the intracellular pathways that could be activated following the binding and might be potential IL-8 regulators, following a literature search, we chose to investigate the expression of nuclear factor-kappa B (NFκB), c-Jun and c-Fos, p38 mitogen-activated protein kinase and extracellular signal-regulated kinase (ERK3/MAPK6) in differentiated F11 cells following paclitaxel treatment. We found that treatment with paclitaxel (10nM) for 24 hours induced the activation of the transcription factor NFκB (Figure 3A and F), significantly increased the expression of the phosphorylated forms of Jun and c-Fos (Figures 3B, C and F), which are the two canonical activator protein 1 (AP-1) transcription factors(29), and of the p38 mitogen-activated protein kinase (MAPK) (Figure 3D and F), while it did not affect the expression of another MAPK family member, p-ERK3/MAPK6 (Figure 3E and F). To further understand the timeline of the effects mediated by C5aR1 activation by paclitaxel and C5a, we performed time-course experiments and evaluated the phosphorylation of the analyzed proteins at different timepoints (i.e. at 15, 30, 60 and 90 minutes of treatment). Interestingly, our results showed that all the analyzed proteins that were activated by both paclitaxel and C5a treatments (p-NFKB, p-C-Jun, p-C-Fos and p-P38) became phosphorylated within 60-90 minutes of treatment (Figure S3A). Notably, the co-administration of a C5aR1 inhibitor, DF3966A (formerly referred to as DF3016A)(11) at 10µM for 24 hours, completely counteracted paclitaxel-induced upregulation of p-NFκB, p-c-Fos and p-p38 (Figures 3A, C, D and F), while it did not affect p-Jun and ERK3/MAPK6 expression (Figure 3B, E and F). 

These data suggest that C5aR1 is crucial for the paclitaxel-induced activation of NFkB/p38 pathway and c-Fos, which are known to be involved in the downstream activation of the IL-8 signaling.

- C5aR1 inhibition modulates paclitaxel-induced alteration of key neuronal plasticity factors 

Starting from the results obtained in our previous study showing that CXCL1 (i.e., murine IL-8) mediates paclitaxel-induced neuronal toxicity in F11 sensory neurons(1), and from our data indicating that the activation of C5aR1 by paclitaxel is upstream of IL-8/CXCL1 pathway, we then investigated whether and how the activation and the inhibition of C5aR1 would affect the expression of key pain markers in F11 cells. With this aim, we first assessed the protein expression of C5aR1 (Figure S4A) in differentiated F11 cell line, and then we treated these cells for 24 hours with paclitaxel (10nM) or C5a (10nM), the natural ligand of C5aR1. Western blot analysis showed that treatment with paclitaxel or C5a increased the expression of p-cortactin, which modulates terminal axonal arborization and synaptic plasticity(12), and of p-FAK, which is responsible for microtubule stabilization(13), suggesting that the two stimuli similarly affect neuronal plasticity (Figures 4A and B). Also p-JAK2 and p-STAT3 protein levels were increased by paclitaxel as well as by C5a (Figures 4C and D) compared to control condition, demonstrating that both stimuli can modulate the JAK-STAT pathway, which is involved in neuropathic pain and neuronal plasticity(14). To further understand the timeline of the effects mediated by C5aR1 activation by paclitaxel and C5a, also in this case we performed time-course experiments and evaluated the phosphorylation of the analyzed proteins at different timepoints (i.e. at 15, 30, 60 and 90 minutes of treatment). Our results showed that the analyzed proteins that were activated by both paclitaxel and C5a treatments (p-FAK, p-CORT and p-JAK) became phosphorylated after 60-90 minutes of treatment, thus indicating them as direct C5aR1-mediated responses (Figure S3B). Notably in fact, both paclitaxel and C5a treatment stimulated CXCL1 mRNA expression in F11 cells compared to control condition at 20 hours (Figure S4B) and paclitaxel increased CXCL1 protein levels at 24 hours in the same cells(1), thus indicating that the activation of CXCL1-mediated pathway is subsequent to these responses that are - at least initially - induced by C5aR1 activation.

To assess whether these effects were indeed mediated by C5aR1 and to investigate the potential therapeutic effects of the inhibition of C5aR1, we then treated F11 cells with paclitaxel in combination with an antibody against C5aR1 (C5aR1 Ab, 50nM). C5aR1 Ab significantly counteracted paclitaxel-induced upregulation of p-Fak and p-JAK2 protein levels (Figure S5), confirming the key role of C5aR1 in the mediation of paclitaxel-induced neurotoxicity and leading us to explore the potential effects of the pharmacological treatment with a C5aR1 antagonist, DF3966A. Western blot analyses showed that treatment with DF3966A (10µM) counteracted paclitaxel-induced increase of p-Fak and p-cortactin (Figure 4E), restoring their expression to control values, and also markedly reduced paclitaxel-induced increment of p-JAK2 and p-STAT3 protein levels (Figure 4F). Moreover, real-time PCR analysis showed that DF3966A also reduced paclitaxel-induced increased expression of transient receptor potential (TRP) cation channels TRPV1 and TRPV4 (Figure 4G), which are crucial mediators of thermal, chemical, and mechanical stimuli in nociception(15). 

Altogether these data clearly demonstrate that the activation of C5aR1 is an upstream event to the molecular alterations that we previously observed in F11 cells following stimulation with paclitaxel and CXCL1(1), suggesting that CXCL1/IL-8 acts later in time to further sustain and make the alterations chronic. Notably, the inhibition of C5aR1 can effectively counteract paclitaxel downstream neuropathological effects, supporting the concept that direct binding and activation of C5aR1 is a key mechanism and a potential therapeutic target for paclitaxel-induced neurotoxicity. 

- C5aR1 inhibition counteracts paclitaxel-induced functional and structural alterations in rat DRG 

In vivo, dorsal root ganglia (DRG) are among the neural tissues that are most vulnerable to and directly affected by the toxic action of anti-cancer drugs, as they are devoid of a complex vascular-nerve barrier able to prevent the diffusion of chemotherapeutics(16). Thus, to further investigate the neuropathological mechanisms underlying paclitaxel-induced neurotoxicity and, especially, the role of C5aR1 in this context, we treated rat DRG primary cultures with paclitaxel (10nM) or C5a (100 nM) in the absence or presence of a C5aR1 inhibitor, DF3966A (1µM), and performed electrophysiological measurements. Compared to the untreated group (CTR), DRG neurons exposed to paclitaxel showed a significantly increased cell action potential firing rate in the short term (1 minute and 30 seconds exposure to the compounds), followed by an electrical activity drop at longer (5 minutes) exposure time (-83.51% vs control) that was due to the inactivation of the voltage-gated channels (Figures 5A, B and F). Likewise, the exposure to C5a immediately increased the firing rate with a significant reduction of the electrical activity at a longer time of exposure (Figures 5A-D and 5G), revealing that paclitaxel and C5a similarly and rapidly alter DRG primary neurons electrophysiological behaviors. The rapidity (few minutes) of the effects induced by both stimuli on DRG electrical activity further strengthens the idea that it is the direct binding of paclitaxel or C5a to C5aR1 that mediates the rapid onset of these alterations rather than an indirect modulation of the complement system.

Treatment with DF3966A completely restored the altered electrical activity induced by both short and long exposures to paclitaxel (Figures 5C and F) and counteracted the C5a-induced electrical activity increment at short time points and blocked its reduction (at about 35%) at longer exposure times (Figures 5E and G). These data clearly demonstrate the crucial role of C5aR1 in the neuropathological mechanisms underlying paclitaxel-induced CIPN and the protective action of C5aR1 inhibition on paclitaxel-treated DRG.

- Paclitaxel, and not its vehicle, is responsible for anaphylactoid reactions in vivo

In the literature, some studies have associated HSRs to paclitaxel nonionic emulsifier vehicle, Cremophor EL (polyoxyethylated castor oil)(17,18), while others suggested a direct effect of paclitaxel, and not of its diluents(19,20), leaving still open the debate on which are the factors and the mechanisms responsible for the anaphylactoid reactions in patients undergoing treatment. Thus, before testing our hypothesis on the potential role of C5aR1, the first mandatory step was to clarify which one among paclitaxel and its vehicle (Cremophor EL) is responsible for inducing the HSRs. Balb/c mice were thus divided in 4 groups and treated with paclitaxel in Cremophor EL (20 mg/kg and 0.1mL/Kg, iv), paclitaxel in DMSO/PEG/TWEEN (20 mg/kg and 0.1mL/Kg, iv), and Cremophor EL (0.1mL/Kg, iv) or DMSO/PEG/TWEEN (0.1mL/Kg, iv) alone. Mice receiving paclitaxel in Cremophor EL or in DMSO/PEG/TWEEN had acute HSRs that were more serious displaying greater vascular leakage (hyper-permeability) and plasma extravasation compared to mice treated with both vehicles alone (Figures S7A and B), thus confirming the crucial role of paclitaxel per se in inducing HSRs

- Pharmacokinetic studies

Pharmacokinetic experiments were performed in Balb/c mice food deprived from 18h before starting oral treatment. DF3966A was dissolved in water (14.7 mg/10 ml) and given in a single oral (by gavage) and intravenous administration at the dose of 14.7 mg/Kg. Blood samples from 3 mice/time were collected at 10 min, 1, 2, 4, 8 and 24h after treatment. 

- Pharmacokinetic profile

DF3966A was rapidly absorbed after oral administration, and the maximum concentration (Cmax) in plasma (35.36 μg/mL) was reached 3h post administration. Overall systemic exposure (AUC) was 233.23 μg/ml/h and the t1/2 was 3.72h. Intravenous administration of DF3966A showed a systemic clearance of 1.05 mL/min/kg. The bioavailability of DF3966A was calculated as 100%. The volume of distribution at steady state was low with respect to total body water (0.33 L/kg) and the t1/2 was 3.63h after dosing. DF3966A was well absorbed by the oral route and gradually eliminated from mouse plasma with an overall profile suitable for repeated chronic administration.

Diagram

Description automatically generated

Figure S1

Diagram, schematic

Description automatically generated

Figure S2

A picture containing text, metalware

Description automatically generated

Figure S3

Chart

Description automatically generated

 Figure S4

Figure S5

Diagram

Description automatically generated

Figure S6

Logo

Description automatically generated

Figure S7

Supplementary Figure Legends

Figure S1. A) Measurement of steady-state binding levels of paclitaxel on immobilized C5aR1. B) Specificity of the monoclonal anti-C5aR1 antibody. C) Co-injection of anti-C5aR1 antibody and C5aR1 on immobilized C5a. D) Modulation of C5aR1 activity by paclitaxel in single concentration and dose-response experiments. 

Figure S2. A) Overlay of SPR sensorgrams showing interaction of different concentrations of docetaxel with immobilized C5AR1 (from 15.625 μM to 250 μM). B) Co-injection of docetaxel and C5aR1 on immobilized C5a and relative IC50. C) Co-injection of docetaxel and C5aR1 on immobilized C5a des Arg and relative IC50. D) Co-injection of docetaxel and C5aR1 on immobilized CD88 antibody and relative IC50.

Figure S3. Western blotting analysis and relative quantification for the tested protein upon C5a and Paclitaxel (PAC, 10nM) at different time points (15-90 minutes): A) Protein levels of p-NFKB, p-C-Jun. p-FOS and p-P38. B) Protein levels of p-FAK, p-CORT, p-JAK and p-MAPK.  *P<0.05 vs CTR. Data are mean ± SEM of n=3 different experiments.

Figure S4. A) C5aR1 expression in differentiated F11 cells. B) CXCL1 expression following paclitaxel or C5a treatment in F11 cells.

Figure S5. Quantification of p-FAK and p-JAK2 expression in control (CTR), paclitaxel (PAC), IgG2B Antibody (Ab), PAC+ IgG2B Ab, C5aR Ab, PAC+C5aR Ab-treated F11 cells evaluated by western blot analysis. **P<0.005; *P<0.05 vs CTR; +P<0.05 ++ p<0.005 vs PAC. Data are mean ± SEM of n=3 different experiments.

Figure S6. Cold and mechanical allodynia in C5ar1-/- mice.  Time course of the effect of paclitaxel (PAC) on cold (A) and mechanical (B) allodynia in wild-type (WT) and C5ar1-/- mice. *P<0.05 vs WT. Data are shown as mean ± SEM of 10 animals per group. 

Figure S7. Effect of paclitaxel (PAC) in DMSO/PEG/TWEEN (20 mg/kg and 0.1mL/Kg, iv) or DMSO/PEG/TWEEN alone (0.1mL/Kg, iv) and paclitaxel in Cremophor EL (20 mg/kg and 0.1mL/Kg, iv) or Cremophor EL alone (0.1mL/Kg, iv) on mouse vascular permeability (A) and plasma extravasation (B). **P<0.005 and *P<0.05 vs PEG/TWEEN; +P<0.05 vs Cremophor. Data are shown as mean ± SEM of 10 animals per group. 

References

1. 	Brandolini L, Elisabetta B, Adelchi RP, Roberto R, Loredana C, Andrea A, et al. CXCR1/2 pathways in paclitaxel-induced neuropathic pain. Oncotarget. 2017 Feb 20;8(14):23188–201. 

2. 	Hsu MH, Wang M, Browning DD, Mukaida N, Ye RD. NF-κB Activation Is Required for C5a-Induced Interleukin-8 Gene Expression in Mononuclear Cells. Blood. 1999 May 15;93(10):3241–9. 

3. 	Wang L, Han G, Wang R, Chen G, Xu R, Xiao H, et al. Regulation of IL-8 production by complement-activated product, C5a, in vitro and in vivo during sepsis. Clinical Immunology. 2010 Oct 1;137(1):157–65. 

4. 	Cao Q, McIsaac SM, Stadnyk AW. Human colonic epithelial cells detect and respond to C5a via apically expressed C5aR through the ERK pathway. Am J Physiol Cell Physiol. 2012 Jun 15;302(12):C1731-1740. 

5. 	Bezzerri V, Borgatti M, Finotti A, Tamanini A, Gambari R, Cabrini G. Mapping the transcriptional machinery of the IL-8 gene in human bronchial epithelial cells. J Immunol. 2011 Dec 1;187(11):6069–81. 

6. 	Yang HT, Cohen P, Rousseau S. IL-1beta-stimulated activation of ERK1/2 and p38alpha MAPK mediates the transcriptional up-regulation of IL-6, IL-8 and GRO-alpha in HeLa cells. Cell Signal. 2008 Feb;20(2):375–80. 

7. 	Namba S, Nakano R, Kitanaka T, Kitanaka N, Nakayama T, Sugiya H. ERK2 and JNK1 contribute to TNF-α-induced IL-8 expression in synovial fibroblasts. PLOS ONE. 2017 Aug 14;12(8):e0182923. 

8. 	Valenty LM, Longo CM, Horzempa C, Ambesi A, McKeown-Longo PJ. TLR4 Ligands Selectively Synergize to Induce Expression of IL-8. Adv Wound Care (New Rochelle). 2017 Oct 1;6(10):309–19. 

9. 	Cho JS, Han IH, Lee HR, Lee HM. Prostaglandin E2 Induces IL-6 and IL-8 Production by the EP Receptors/Akt/NF-κB Pathways in Nasal Polyp-Derived Fibroblasts. Allergy Asthma Immunol Res. 2014 Sep;6(5):449–57. 

10. 	Aso H, Ito S, Mori A, Morioka M, Suganuma N, Kondo M, et al. Prostaglandin E2 enhances interleukin-8 production via EP4 receptor in human pulmonary microvascular endothelial cells. Am J Physiol Lung Cell Mol Physiol. 2012 Jan 15;302(2):L266-273. 

11. 	Brandolini L, Grannonico M, Bianchini G, Colanardi A, Sebastiani P, Paladini A, et al. The Novel C5aR Antagonist DF3016A Protects Neurons Against Ischemic Neuroinflammatory Injury. Neurotox Res. 2019 Jul;36(1):163–74. 

12. 	Alicea D, Perez M, Maldonado C, Dominicci-Cotto C, Marie B. Cortactin Is a Regulator of Activity-Dependent Synaptic Plasticity Controlled by Wingless. J Neurosci. 2017 Feb 22;37(8):2203–15. 

13. 	Palazzo AF, Eng CH, Schlaepfer DD, Marcantonio EE, Gundersen GG. Localized stabilization of microtubules by integrin- and FAK-facilitated Rho signaling. Science. 2004 Feb 6;303(5659):836–9. 

14. 	Simon LS, Taylor PC, Choy EH, Sebba A, Quebe A, Knopp KL, et al. The Jak/STAT pathway: A focus on pain in rheumatoid arthritis. Seminars in Arthritis and Rheumatism. 2021 Feb 1;51(1):278–84. 

15. 	Dai Y. TRPs and pain. Semin Immunopathol. 2016 May;38(3):277–91. 

16. 	Quintão NLM, Santin JR, Stoeberl LC, Corrêa TP, Melato J, Costa R. Pharmacological Treatment of Chemotherapy-Induced Neuropathic Pain: PPARγ Agonists as a Promising Tool. Frontiers in Neuroscience. 2019;13:907. 

17. 	Szebeni J, Alving CR, Muggia FM. Complement Activation by Cremophor EL as a Possible Contributor to Hypersensitivity to Paclitaxel: an In Vitro Study. JNCI: Journal of the National Cancer Institute. 1998 Feb 18;90(4):300–6. 

18. 	Picard M, Castells MC. Re-visiting Hypersensitivity Reactions to Taxanes: A Comprehensive Review. Clin Rev Allergy Immunol. 2015 Oct;49(2):177–91. 

19. 	Nolte H, Carstensen H, Hertz H. VM-26 (teniposide)-induced hypersensitivity and degranulation of basophils in children. Am J Pediatr Hematol Oncol. 1988;10(4):308–12. 

20. 	Essayan DM, Kagey-Sobotka A, Colarusso PJ, Lichtenstein LM, Ozols RF, King ED. Successful parenteral desensitization to paclitaxel. J Allergy Clin Immunol. 1996 Jan;97(1 Pt 1):42–6.
